# Blue Light Emitting Diode Suppresses Sarcoma Cell Proliferation via the Endogenous Apoptotic Pathway Without Damaging Normal Cells

**DOI:** 10.1002/cam4.70770

**Published:** 2025-03-24

**Authors:** Shinji Kawaguchi, Toshihiko Nishisho, Shunichi Toki, Makoto Takeuchi, Shunsuke Tamaki, Koichi Sairyo

**Affiliations:** ^1^ Department of Orthopedics, Institute of Biomedical Sciences Tokushima University Graduate School Tokushima Japan

**Keywords:** blue light, endogenous apoptosis, normal human dermal cell, osteosarcoma, reactive oxygen species, soft tissue sarcoma

## Abstract

**Background:**

The development of novel therapies for sarcoma is urgently required because most sarcomas are refractory to adjuvant therapy and the treatment options are limited. Although antitumor effects of blue light (BL) have been reported for several malignant tumors, its impact on various sarcomas remains unknown. In this study, we examined the antitumor effects of BL on several kinds of bone and soft tissue sarcomas.

**Methods:**

We used human osteosarcoma, undifferentiated pleomorphic sarcoma, liposarcoma, and myxofibrosarcoma cell lines, as well as a human dermal fibroblast cell line as normal cells. We continuously irradiated these cells with BL in an incubator.

**Results:**

BL inhibited cell proliferation in sarcoma cells, but hardly affected normal cells. BL increased intracellular reactive oxygen species (ROS) levels in sarcoma cells, but not in normal cells. Interestingly, oxidative stress occurred after BL irradiation in both sarcoma and normal cells. In addition, apoptosis, autophagy, and mitochondrial dysfunction, which were induced by ROS, occurred in sarcoma cells. In undifferentiated pleomorphic sarcoma cells, BL caused cell death through activation of the endogenous apoptotic pathway, and autophagy counteracted the apoptosis.

**Conclusion:**

Our results indicate that BL might specifically kill sarcoma cells without injuring normal cells and may become a new treatment option for sarcoma.

## 1 Introduction

The prevalence of bone and soft tissue sarcomas is about 5 cases per 100,000 persons. They are thus very infrequent, accounting for only 1% of all carcinomas. Furthermore, bone and soft tissue sarcomas are classified into about 70 subtypes, each characterized by a distinct morphology, and many histological types are extremely rare [1, 2]. In principle, bone and soft tissue sarcomas are treated via extensive resection [1]. Because the resection requires the removal of the surrounding normal tissue, the patients' activities of daily living (ADLs) are often postoperatively reduced. Although combination chemotherapy and radiation therapy are also considered, these treatments have limited effectiveness in almost all sarcoma cases. Therefore, the development of new adjuvant therapies for sarcomas is desirable. However, their rarity and diversity complicate advancements in systematic research, and the development of new treatments is lagging.

Visible light affects our bodies. For example, red light and near‐infrared light (600–750 nm) promote wound healing and pain relief in joint diseases [3]. In addition, blue light (BL, 407–420 nm) irradiation has been reported to control bacterial infection [4]. Recent years have seen the publication of multiple reports showing that visible BL (430–490 nm) has antitumor effects in various types of cancer cells, including malignant melanoma (a superficial cancer) [5], colon cancer [6], malignant lymphoma [7], pancreatic cancer [8], and leukemia [9]. For sarcoma, an antitumor effect of blue LED light on osteosarcoma was reported in 2021 [10]. Although we reported an antitumor effect of BL on synovial sarcoma in 2023 [11], no study has investigated the antitumor effects of blue LED light on soft tissue sarcomas. There are scattered reports on various carcinomas, and we believe that BL will become a novel therapy for malignant tumors.

Blue LEDs are considered to exert phototoxic and antiproliferative effects through the generation of intracellular reactive oxygen species (ROS) [8]. In osteosarcoma, blue LED has been reported to induce ROS‐dependent autophagic cell death [10]. In addition, our results showed that blue LED inhibits the cell proliferation of malignant melanoma, malignant lymphoma, and synovial sarcoma via apoptosis. Thus, the mechanisms underlying the interaction of blue LED irradiation with molecular targets and the promotion of cell damage are unclear.

Despite the wide variety of sarcomas, there are no reports on the antitumor effects of blue LED light other than for osteosarcoma and synovial sarcoma. Therefore, in this study, we investigated whether blue LED irradiation has antiproliferative and apoptotic effects in various sarcoma cell lines, including osteosarcoma. The aim of this study is to provide a unified view of the mechanism of the antitumor effect of blue LED light on sarcoma cells.

## 2 Methods

### 2.1 Cell Culture

In these experiments, we used the following human sarcoma cell lines: osteosarcoma (U‐2 OS), undifferentiated pleomorphic sarcoma (NCC‐UPS1‐C1), liposarcoma (SW872), and myxofibrosarcoma (NCC‐MFS4‐C1). We also used an NHDF cell line as normal cells for comparison with the sarcoma groups. U‐2 OS was purchased from the European Collection of Authenticated Cell Cultures, and SW872 was obtained from the American Type Culture Collection. NCC‐UPS1‐C1 and NCC‐MFS4‐C1 were established at the National Cancer Center in Japan and transferred to our laboratory for this research [12, 13]. NHDF was purchased from PromoCell. As soon as cell lines were received from these cell banks, they were passed on and stored. All cells were cultured in Dulbecco's modified Eagle medium (Sigma‐Aldrich) supplemented with 10% fetal bovine serum (FBS; Sigma‐Aldrich) and penicillin/streptomycin (Sigma‐Aldrich) in a humidified atmosphere of 5% CO_2_ at 37°C, as in our previous study [11]. The chemical reagents used in this study are listed in Table S1.

### 2.2 Light Irradiation

Teleopto LED array systems (LEDA‐X LED Array with LAD‐1 driver; Amuza) were used for each light irradiation. This LED device is designed for multi‐well plates and is thus equipped with 96 light sources for irradiating 96 wells (Figure S1). For 6‐well plates, the irradiation is largely uniform with negligible differences, although errors may occur within a single well due to the distance from the light source. The LED light intensity was 0.1 mW/cm^2^, 0.6 mW/cm^2^, and 1.3 mW/cm^2^ of blue (peak at 470 nm), green (peak at 525 nm), and red (peak at 630 nm) light. The light intensity was measured with a Light Power Meter (LPM‐100; Amuza). Cell culture and LED light irradiation were performed in a 5% CO_2_ incubator at 37°C in the dark. The cells received light irradiation from the bottom of a multi‐well plate. Non‐irradiated controls were also cultured in the incubator and not exposed to light. The LED light intensity used in this experiment (0.1 mW/cm^2^, 0.6 mW/cm^2^, and 1.3 mW/cm^2^) did not increase the temperature of the culture medium in a multi‐well plate nearly as much as in the non‐irradiated group (0 mW/cm^2^) (Figure S2a). After cell seeding and 24 h of culture, the plate was immediately placed in the LED device and the irradiation was started. Except in the colony formation assay, the cells were cultured for 12, 24, 48, or 72 h with continuous irradiation. Immediately after irradiation, the samples were used for further study. The light doses at each light intensity are shown separately in Figure S2b. NHDF cells and sarcoma cells irradiated with LED light were used in the experiments. For comparison, NHDF cells and sarcoma cells that were not irradiated with LED light were used as a control group.

### 2.3 Cell Viability Assay

We used a CCK‐8 kit (Dojindo) to measure the proliferation of the sarcoma cells and the dermal fibroblast cells, as described previously [11]. Briefly, a total of 2.5–10.0 × 10^3^ cells in a volume of 100 μL per well were cultured in at least triplicate wells in a 96‐well plate (Thermo Fisher Scientific). The cells were incubated overnight until they attached and were then exposed to light of various intensities. The cells were incubated with light of each wavelength for 24, 48, or 72 h. Immediately after irradiation, CCK‐8 reagent (10 μL) was added to 90 μL DMEM to generate a working solution; 100 μL of this solution was added to each well and was left to incubate for 1 h. The absorbance was measured at 450 nm with a microplate reader (Varioskan Flash; Thermo Fisher Scientific). We performed this assay at 0, 24, 48, and 72 h.

### 2.4 Wound Healing Assay

Sarcoma cells were seeded on a culture insert (ibidi culture‐insert 2 well; ibidi GmbH) at a density of 3.0 × 10^5^ cells/mL. After the cells were incubated overnight until attachment, the culture insert was removed and continuously irradiated by BL (1.3 mW/cm^2^) for the indicated times. Wound areas were photographed, and the wound width rate was calculated under a light microscope (Nikon) as follows: wound width rate = width of the wound at *n* h/width of the wound at 0 h.

### 2.5 Colony Formation Assay

Sarcoma cells were seeded in 6‐well plates (Thermo Fisher Scientific) at a density of 1 × 10^3^ cells per well. BL irradiation (1.3 mW/cm^2^) was started immediately after cell attachment. The non‐irradiated group and the groups irradiated with BL for 1 or 6 h were then cultured without light in an incubator for 14 days until colonies formed. Finally, the cells were fixed and stained with a mixture of 6.0% glutaraldehyde and 0.5% crystal violet (Sigma‐Aldrich) as previously reported [14] and then photographed.

### 2.6 Transwell Invasion Assay

Matrigel‐coated inserts that fit into 24‐well chambers (Corning) were used for the invasion assay. The cells were seeded in the upper chamber at a density of 4.0 × 10^4^ cells/0.5 mL in serum‐free medium. Then, 0.75 mL 10% FBS‐supplemented medium was added to the lower chamber. The cells were allowed to invade under BL irradiation. After the irradiation, non‐invasive cells were gently removed from the surface of the chamber with cotton swabs. Next, the invaded cells on the chamber bottom were fixed and stained using Diff‐Quik solutions (Sysmex). After the filter was dried, the invaded cells were imaged and counted under a light microscope.

### 2.7 Apoptosis Assay

A Dead Cell Apoptosis Kit with Annexin V for Flow Cytometry (Thermo Fisher Scientific) was used to measure cell apoptosis according to the manufacturer's instructions. Briefly, cells were seeded in a 6‐well plate at a density of 0.5–1.0 × 10^5^ cells per well and irradiated with BL (1.3 mW/cm^2^) for the indicated times. After the irradiation, the cells were harvested and washed twice with PBS. The obtained cells were suspended in 500 μL Annexin binding buffer containing Annexin V‐FITC and propidium iodide and incubated for 15 min in the dark at room temperature. Fluorescence was measured on a flow cytometer (BD FACSVerse; Becton Dickinson).

### 2.8 Quantitative PCR Analysis

Total RNA was extracted using the RNeasy Kit (Qiagen). RNA from each sample was used to synthesize complementary DNA using the iScript cDNA Synthesis Kit (Bio‐Rad, Hercules, CA) according to the manufacturer's protocol. For quantitative PCR (qPCR), we used the Power SYBR Green Master Mix (Thermo Fisher Scientific) and the results were normalized to 18S ribosomal RNA. The primers used are listed in Table S2.

### 2.9 Protein Extraction and Western Blotting

Cells were harvested with a scraper and lysed in RIPA buffer supplemented with protease and phosphatase inhibitors (Thermo Fisher Scientific). After incubation on ice for 30 min, the lysates were centrifuged at 15,000 rpm for 15 min at 4°C, and then the supernatant was moved to a new tube for analysis of the protein concentrations using the BCA Protein Assay Kit (Takara). The collected proteins were subjected to 8%, 10%, or 15% sodium dodecyl sulfate‐polyacrylamide gel electrophoresis and transferred to 0.45 μm PVDF membranes (Millipore, MA). After blocking, the membranes were incubated at 1:1000 with the following primary antibodies: poly (ADP‐ribose) polymerase (PARP) (#9542; Cell Signaling Technology [CST]), light chain 3B (LC3B) (#2775; CST), caspase‐3 (#9662; CST), cytochrome c (#4272; CST), BCL‐2 (#4223; CST), BAX (#2772; CST), and α/β tubulin (#2148; CST). The membranes were washed with Tris‐buffered saline with 0.1% Tween buffer and incubated with diluted anti‐rabbit IgG horseradish peroxidase‐linked secondary antibody (1:3000, #7074; CST). Blots were visualized using Amersham ECL Prime (Cytiva, Tokyo, Japan). The FUSION FX. EDGE System (Vilber Lourmat) was used for imaging, and protein levels were based on signal intensity.

### 2.10 Determination of ROS

CellROX reagent (Thermo Fisher Scientific) for detecting intracellular (total) ROS and MitoSOX Red reagent (Thermo Fisher Scientific) for detecting mitochondrial ROS were used according to the manufacturer's instructions. Briefly, sarcoma cells were seeded in a 6‐well plate at 2.5 × 10^4^ cells per well and incubated overnight. The cells were then exposed to BL for 48 h. After the irradiation, the cells were treated with CellROX (1 μM) for 1 h or MitoSOX (5 μM) for 30 min. The fluorescence was measured on a flow cytometer.

### 2.11 Determination of the Oxygen Consumption Rate

The oxygen consumption rate (OCR) was measured using a Seahorse XF Cell Mito Stress assay on the Seahorse XF HS Mini Analyzer (Agilent Technologies) according to the manufacturer's instructions. Briefly, cells were seeded and incubated overnight. Then, the cells were exposed to BL for 48 h. Before measurement, plates were equilibrated in a CO_2_‐free incubator at 37°C for 1 h. Analysis was performed using 1.5 μM oligomycin, 2.0 μM carbonyl cyanide‐4‐(trifluoromethoxy) phenylhydrazone, and 0.5 μM rotenone/antimycin A in accordance with the manufacturer's instructions. Data were analyzed using Seahorse XF Cell Mito Stress Test Report generator software (Agilent Technologies). Data were normalized to the actual cell count using Hoechst 33342 (Thermo Fisher Scientific) staining immediately after OCR recording.

### 2.12 Mitochondrial Transmembrane Potential

The mitochondrial membrane was monitored using the JC‐1 MitoMP Detection Kit (Dojindo) according to the manufacturer's protocol. Briefly, cells were seeded in a 6‐well plate at a density of 2.5 × 10^4^ cells per well and incubated overnight. Then, the sarcoma cells were irradiated with BL (1.3 mW/cm^2^) for 48 h. After the irradiation, the cells were stained with JC‐1 at 37°C for 30 min in the dark. The intensity of the JC‐1 staining was determined using flow cytometry.

### 2.13 Autophagy Assay

The CYTO‐ID Autophagy Detection Kit (Enzo Life Sciences) was used to study autophagy. Cells were seeded in a 6‐well plate at a density of 2.5 × 10^4^ cells per well and incubated overnight. The cells were then irradiated with BL (1.3 mW/cm^2^) for 48 h. Next, the cells were collected and incubated with CYTO‐ID detection reagent for autophagic vesicle staining at 37°C for 30 min in the dark. Subsequently, the stained cells were washed, resuspended, and subjected to flow cytometry.

### 2.14 Statistical Analysis

Plots and statistical tests were generated with GraphPad Prism 9 (GraphPad Software). Data are presented as the mean ± standard error of the mean. All results were confirmed in at least three independent experiments. Comparisons between two groups were conducted by an unpaired Student's t‐test. One‐way analysis of variance followed by Dunnett's multiple comparisons test was used for comparison between different groups. *p* < 0.05 was considered statistically significant.

## 3 Results

### 3.1 Blue LED Irradiation Inhibits Sarcoma Cell Proliferation but Not That of a Normal Cell Line

**FIGURE 1:**
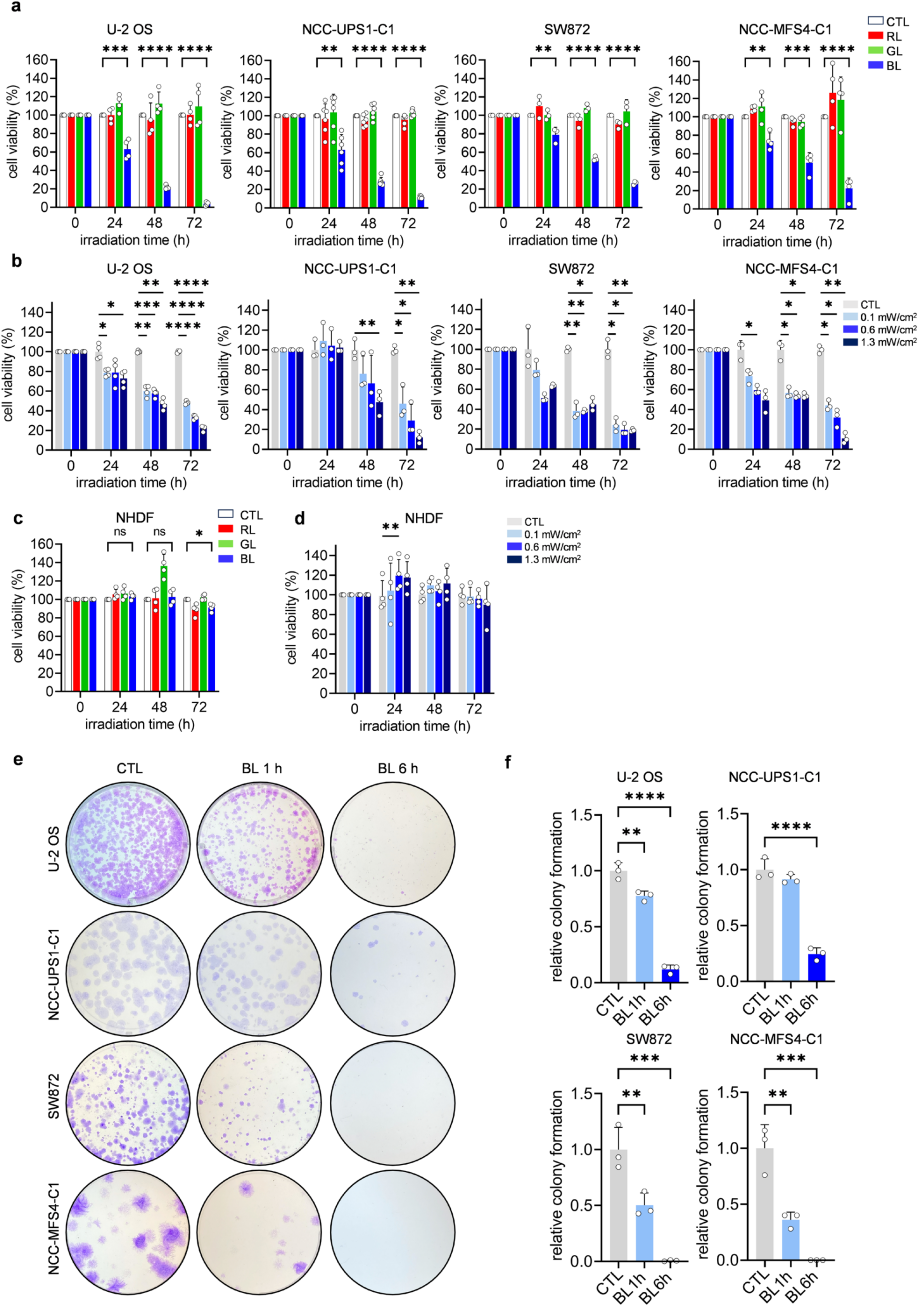
Blue LED light inhibits sarcoma cell proliferation but hardly affects normal cell proliferation. Effects of blue light (BL) on the cell proliferation of sarcoma cells. Cell viability was measured at 24, 48, and 72 h. (a) Cell viability of sarcoma cells irradiated with red light (RL), green light (GL), and BL, as determined using a CCK‐8 assay. Irradiance was administered at 1.3 mW/cm^2^. (b) Cell viability of sarcoma cells examined by BL irradiance with different irradiation times. Irradiances were 0.1 mW/cm^2^, 0.6 mW/cm^2^, and 1.3 mW/cm^2^, respectively. (c, d) Effects of RL, GL, and BL on cell proliferation in normal human skin fibroblasts. (e) Colony formation assay of sarcoma cells incubated with or without BL irradiation (1.3 mW/cm^2^) for 24 h. (f) Quantification of the mean number of colonies formed in each group. Data are expressed as the mean ± standard error of the mean of at least three independent experiments. *p < 0.05, **p < 0.01, ***p < 0.001, ****p < 0.0001.

To determine the effects of irradiation with LED light of various wavelengths on sarcoma cell growth, we used red light, green light, and BL to irradiate U‐2 OS cells (osteosarcoma cell line), NCC‐UPS1‐C1 cells (undifferentiated pleomorphic cell line), SW872 cells (liposarcoma cell line), and NCC‐MFS4‐C1 cells (myxofibrosarcoma cell line). Each LED wavelength light was applied at the same irradiance of 1.3 mW/cm^2^ for up to 72 h. Cell viability was measured at 24, 48, and 72 h from the start of irradiation with a Cell Counting Kit‐8 (CCK8) assay. A time‐dependent decrease in cell viability was observed in the blue LED group for all sarcoma cells, and a marked decrease in cell viability was seen at 72 h irradiation. However, no decrease in cell viability was observed in the red light and green light groups or in the non‐irradiation group (Figure 1a).

Irradiation with blue LED light at different irradiance levels (0.1 mW/cm^2^, 0.6 mW/cm^2^, and 1.3 mW/cm^2^) induced an irradiance‐ and time‐dependent decrease in cell viability, with 1.3 mW/cm^2^ for 48 h resulting in an approximately 50% decrease in the cell viability of all cell lines (Figure 1b). Interestingly, there was little decrease in cell viability with blue LED irradiation in the normal human dermal fibroblast (NHDF) cell line (Figure 1c). Furthermore, there was little decrease in cell viability at the different BL irradiance levels (Figure 1d). Microscopic images showed a decrease in cell number, a rounding of the cell morphology, and an increase in floating cells in the BL‐irradiated groups of sarcomas (Figure S3). The antiproliferative effect of blue LED light on sarcoma cells was confirmed with a colony formation assay as previously reported [12]. The colony‐forming ability of sarcoma cells decreased in a time‐dependent manner after exposure to blue LED light (Figure 1e,f). Furthermore, blue LED irradiation significantly reduced the cell motility of sarcoma cells in a wound healing assay and the cell migration ability of sarcoma cells in a transwell assay (Figures S4 and S5). These results suggested that blue LED light reduced the proliferative ability of sarcoma cells but not that of normal cells.

### 3.2 Blue LED Irradiation Generates ROS in Sarcoma Cells

**FIGURE 2:**
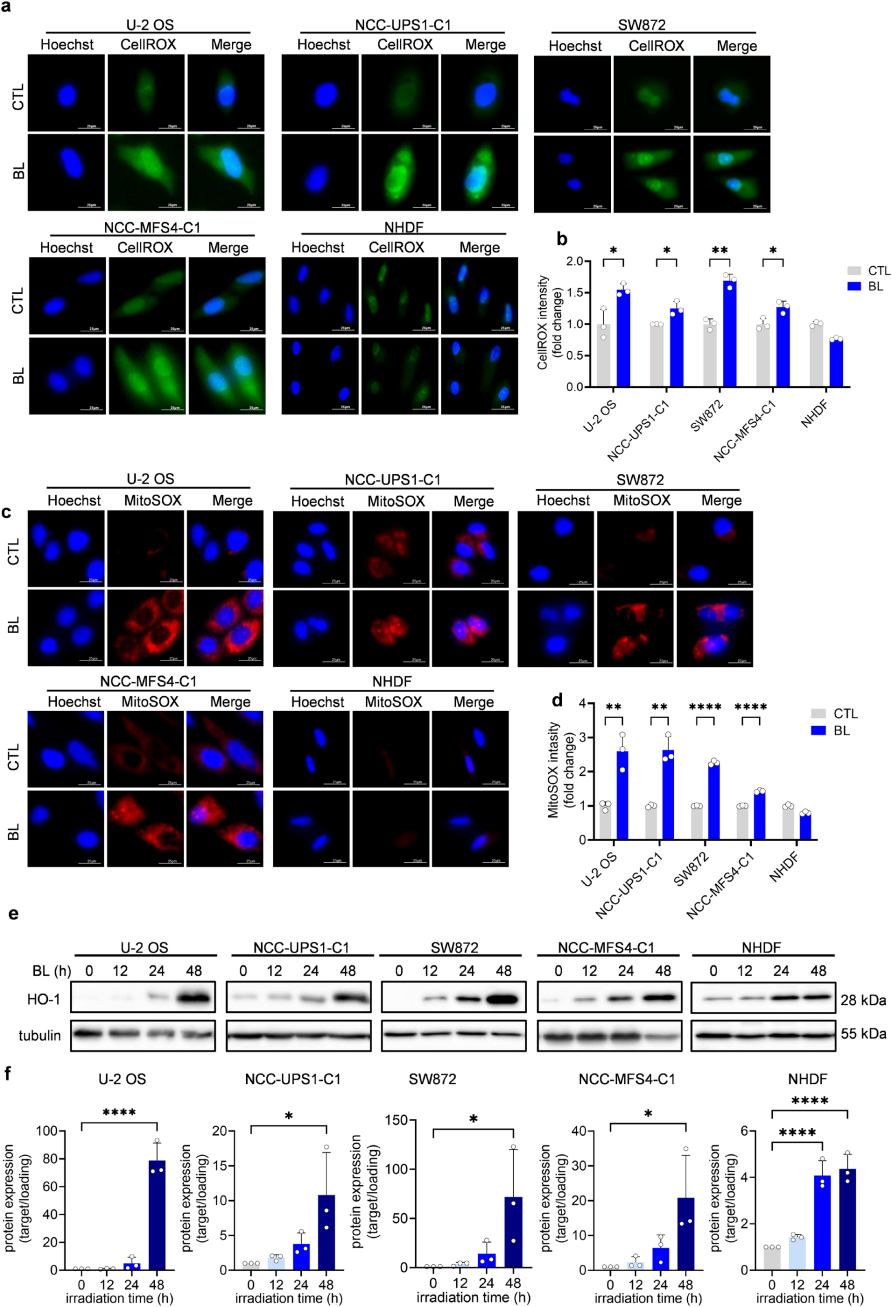
Blue light induces oxidative stress in both sarcoma cells and normal cells but reactive oxygen species induced by blue LED light are scavenged in normal cells. Effects of blue light (BL) on intracellular and mitochondrial reactive oxygen species (ROS) production in sarcoma cells and normal cells. (a, b) Representative fluorescence images and quantitative analysis of intracellular ROS detection with the CellROX assay. Scale bar = 25 μm. (c, d) Representative fluorescence images and quantitative analysis of mitochondrial ROS detection with the MitoSOX assay. Scale bar = 25 μm. (e) Protein levels of HO‐1 in sarcoma and normal cells after exposure to BL irradiation, as determined by western blotting. (f) Quantitative analysis of (e). Data are presented as the mean ± standard error of the mean of three independent experiments. *p < 0.05, **p < 0.01, ***p < 0.001, ****p < 0.0001.

Because the CCK8 assay showed a reduction in cell viability only under BL, we conducted the subsequent experiments using BL at 1.3 mW/cm^2^. The basis of the cellular injury induced by blue LED light is closely related to the generation of intracellular ROS, as has been reported in various cancer cells [6, 7, 8, 9, 10, 11]. The CellROX ROS detection assay was performed to evaluate intracellular ROS induced by blue LED light in sarcoma cells. Fluorescence microscopy showed that green fluorescence was significantly higher in all sarcoma cells in the blue LED‐irradiated group compared to the control group (Figure 2a). In contrast, blue LED irradiation did not enhance green fluorescence in NHDFs, which were used as a comparison. Flow cytometry quantification also showed increased green fluorescence in sarcoma cell lines, but not in NHDFs (Figure 2b). These results indicated that intracellular ROS were increased by blue LED irradiation in all sarcoma cells, consistent with previous studies [6, 7, 8, 9, 10, 11]. Furthermore, in the MitoSOX mitochondrial ROS identification test, blue LED light increased red fluorescence, detected with microscopy and flow cytometry, in sarcoma cells, while NHDFs did not show increased red fluorescence in both the blue LED light irradiated group and the control group (Figure 2c,d). This indicates that blue LED irradiation in sarcoma cells increased intramitochondrial ROS in the same way as in the abovementioned study. Western blotting showed that blue LED irradiation of sarcoma cells increased the expression of HO‐1, a scavenger of the oxidative stress induced by ROS, in a time‐dependent manner. This finding suggested that blue LED caused oxidative damage in sarcoma cells. Interestingly, HO‐1 expression was also significantly higher in NHDFs with blue LED irradiation compared to NHDFs in the control group (Figure 2e,f and Figures S6 and S7). These results revealed that oxidative stress due to ROS occurred in both sarcoma cells and normal skin cells, but that some intracellular ROS removal mechanisms in normal cells eliminated the ROS generated by blue LED irradiation.

### 3.3 Blue LED Irradiation Induces Apoptotic Cell Death

**FIGURE 3:**
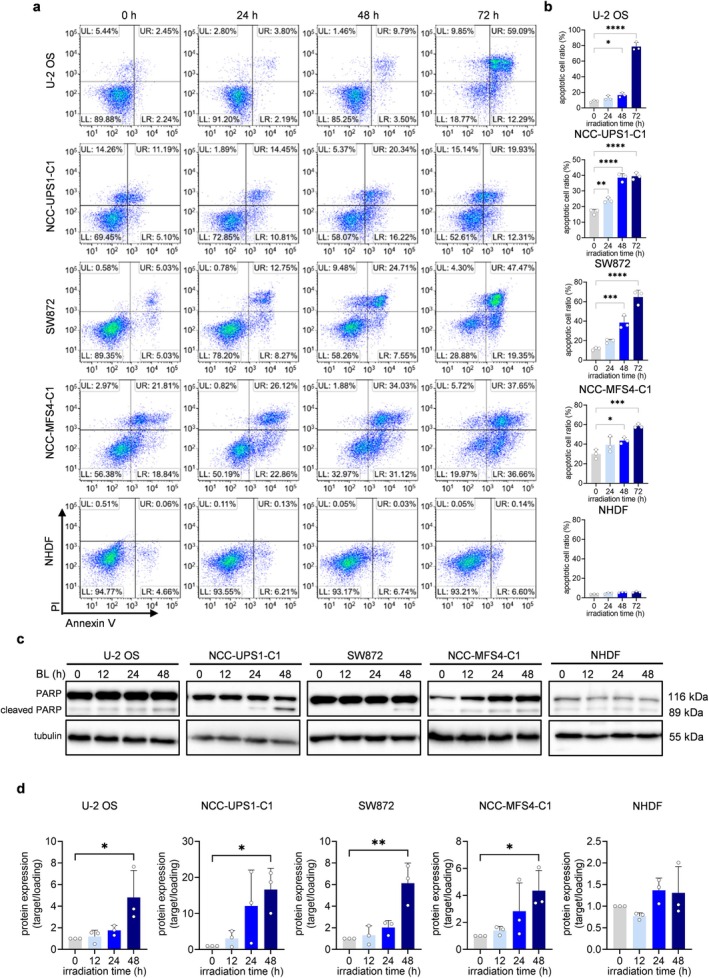
Blue LED light induces apoptosis in sarcoma cells but not in NHDFs. Apoptotic effects of exposure to blue light (BL) on sarcoma cells and normal cells. (a, b) Flow cytometry analysis of the apoptosis in sarcoma cells after BL irradiation. PI, propidium iodide. UR, late apoptosis; LR, early apoptosis; UL, necrosis; LL, viable cells. (c) Western blotting analysis of the expression levels of apoptosis‐related proteins after BL irradiation. (d) Quantitative analysis of (c). Cleaved PARP expression was significantly increased in sarcoma cells irradiated with BL for 48 h. Data are presented as the mean ± standard error of the mean of three independent experiments. *p < 0.05, **p < 0.01, ***p < 0.001, ****p < 0.0001.

We evaluated the mechanism of the sarcoma cell death induced by blue LED irradiation. Specifically, Annexin V and propidium iodide staining were used to identify apoptotic cells. The percentage of apoptotic cells was compared in the group of sarcoma cells irradiated with blue LED for 24, 48, and 72 h and in the non‐irradiated group. In all sarcoma cells, blue LED irradiation significantly increased the number of apoptotic cells in a time‐dependent manner. On the other hand, blue LED irradiation hardly increased the apoptotic cell number in NHDFs (Figures 3a,b and S8). Western blotting was used to examine the expression of the apoptosis marker cleaved PARP in the groups irradiated with blue LED for 12, 24, and 48 h and in the non‐irradiated group. The expression of cleaved PARP was increased in a time‐dependent manner in all sarcoma cells irradiated with blue LED. However, elevated cleaved PARP expression was not observed in the blue light‐irradiated NHDFs (Figure 3c,d and S9). These results suggested that BL might induce apoptosis specifically in sarcoma cells.

### 3.4 Blue LED Irradiation Induces Mitochondrial Damage in Sarcoma Cells

**FIGURE 4:**
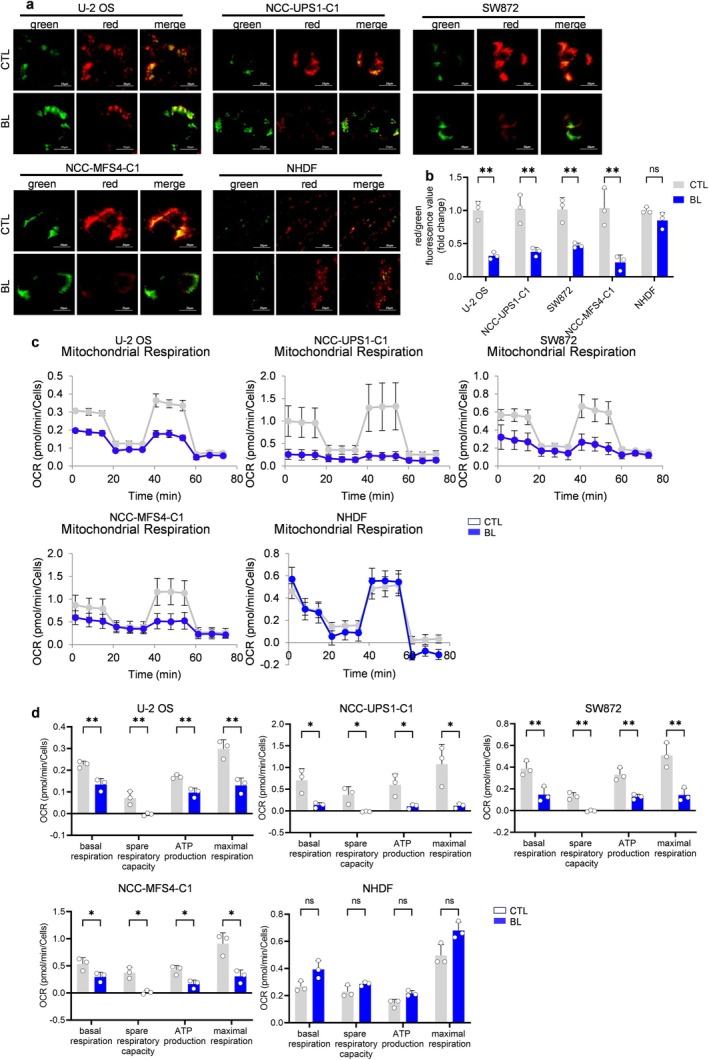
Blue LED light causes mitochondrial dysfunction in sarcoma cells. Blue light (BL) irradiation induces mitochondrial dysfunction. (a, b) Representative fluorescence images and quantitative analysis of the mitochondrial membrane potential with the JC‐1 assay in sarcoma cells and normal cells. Scale bar = 25 μm (*n* = 3, mean ± standard error of the mean). (c, d) The oxygen consumption rate in sarcoma cells and normal cells, as measured with the Seahorse XF Cell Mito Stress assay and Seahorse XF HS Mini Analyzer. Quantification of metabolic parameters (*n* = 3, mean ± standard error of the mean). *p < 0.05, **p < 0.01, ***p < 0.001, ****p < 0.0001.

Next, we examined the effects of blue LED irradiation on mitochondria. When mitochondria are damaged, they release cytochrome c into the cytoplasm, resulting in intrinsic apoptosis via the caspase pathway. The mitochondrial transmembrane potential (ΔΨm) reflects mitochondrial function [15]. Therefore, we attempted to analyze the effect of blue LED on the ΔΨm using fluorescent JC‐1. In Figure 4, red fluorescence represents JC‐1 aggregates that appeared in mitochondria after voltage‐dependent aggregation, while green fluorescence represents JC‐1 monomers that appeared in the cytoplasm after mitochondrial membrane depolarization. Blue LED‐treated sarcoma cells had fewer JC‐1 aggregates (red) and increased JC‐1 monomer (green) compared with the non‐irradiated sarcoma cells (Figure 4a,b). In contrast, blue LED irradiation of NHDFs did not result in increased green fluorescence compared with the non‐irradiated NHDFs, suggesting that it did not cause mitochondrial damage. Furthermore, mitochondrial respiration capacity was examined with the Seahorse XF HS analyzer. The oxygen consumption rate (OCR), a measure of mitochondrial respiration, was significantly suppressed in terms of basal respiration, ATP production, maximal respiration, and spare respiratory capacity with blue LED irradiation for 48 h in sarcoma cells, while normal cells showed no significant differences (Figure 4c,d). These results indicated that blue LED irradiation caused mitochondrial damage in sarcoma cells.

### 3.5 Blue LED Induces Autophagy in Sarcoma Cells

**FIGURE 5:**
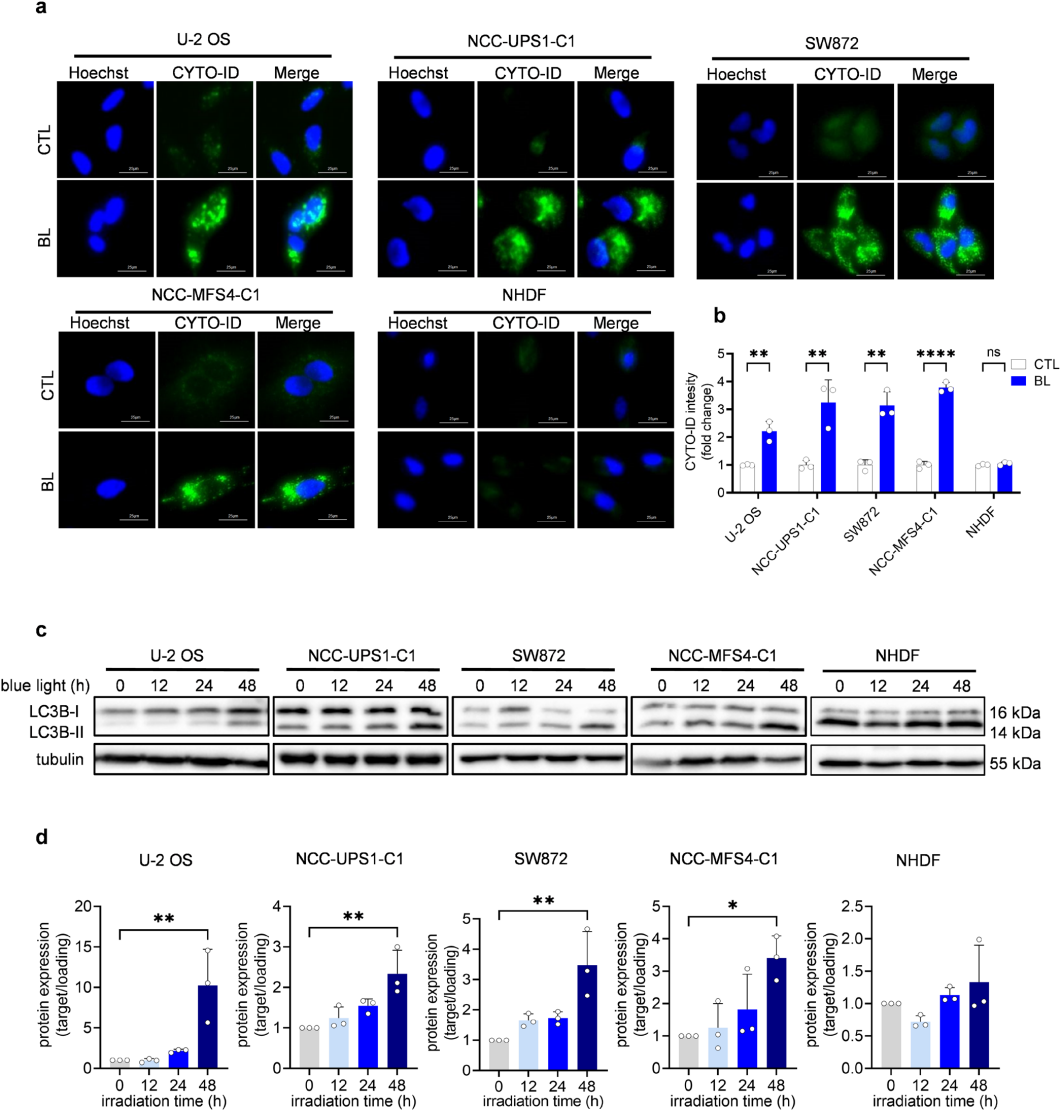
Blue LED light induces autophagy in sarcoma cells but not in NHDFs. Autophagic effects of blue light (BL) on sarcoma cells and normal cells. (a) Representative fluorescence images of autophagy with CYTO‐ID assay after treatment. Scale bar = 25 μm. (b) Quantitative analysis of (a). (c) Western blotting analysis of the expression levels of LC3B‐II after BL irradiation. (d) Quantitative analysis of (c). Data are presented as the mean ± standard error of the mean of three independent experiments. *p < 0.05, **p < 0.01, ****p < 0.0001.

We investigated whether blue LED light could initiate autophagy in sarcoma cell lines. Some studies have reported that ROS can modulate autophagy in cancer cells [16, 17]. The CYTO‐ID autophagy detection assay showed higher green fluorescence in all sarcoma cell lines in the blue LED‐irradiated group compared with the non‐irradiated group but not in NHDFs (Figure 5a). BL also significantly increased green fluorescence in sarcoma cells compared with the non‐irradiated sarcoma cells, as quantified by flow cytometry (Figure 5b). In the sarcoma cells, Western blotting showed that blue LED treatment increased the expression of LC3B‐II, an autophagy marker, in a time‐dependent manner. In contrast, blue LED irradiation did not increase LCB‐II in NHDFs (Figure 5c,d and S10). These results suggested that BL induced autophagy in sarcoma cells.

### 3.6 Blue LED Irradiation Induces ROS‐Dependent Apoptosis and Cell Death in Sarcoma Cells

**FIGURE 6:**
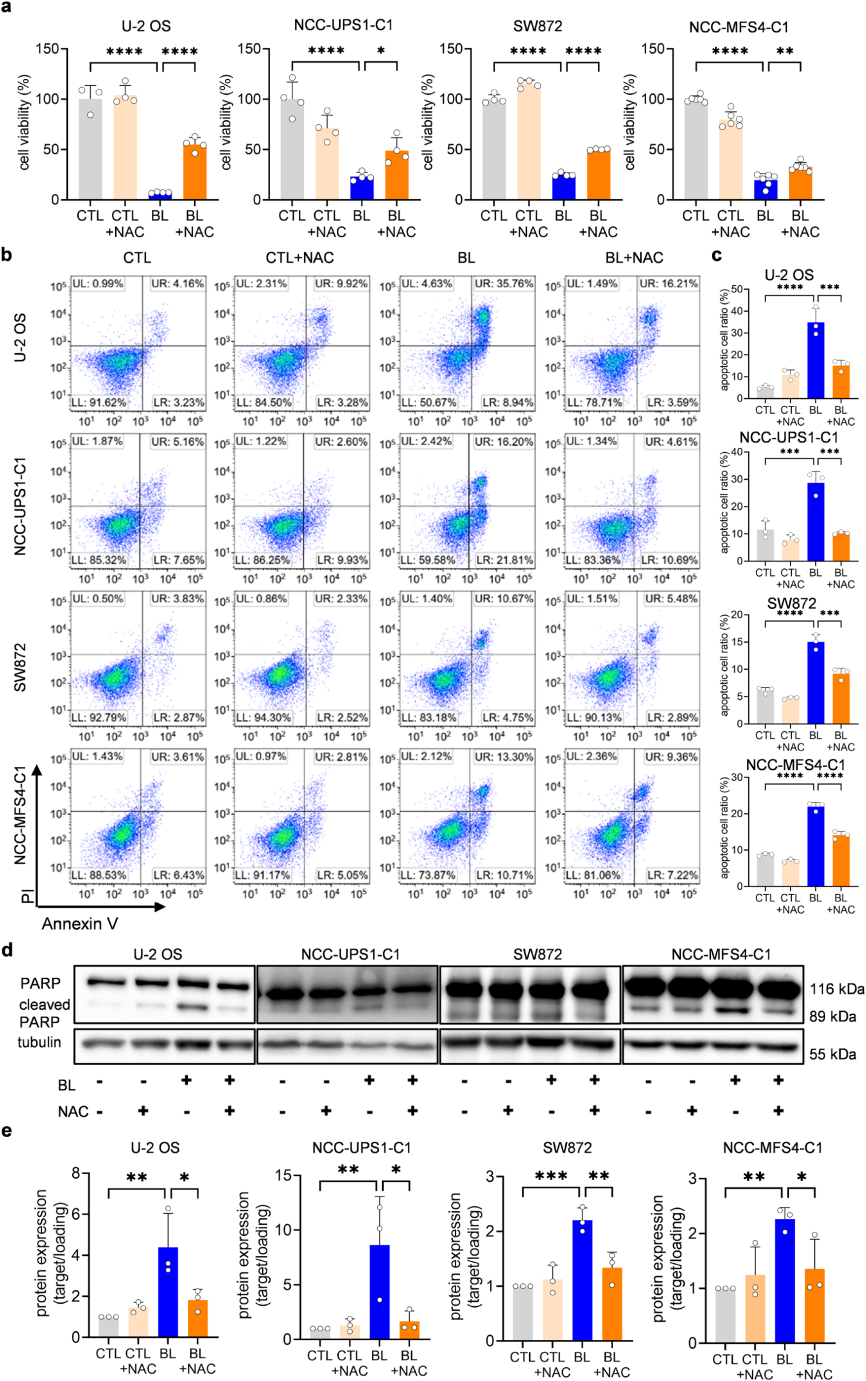
Blue light‐derived reactive oxygen species induce apoptosis in sarcoma cells. The reactive oxygen species scavenger N‐acetyl‐cysteine (NAC, 5 mM) suppresses blue light‐derived apoptosis and increases cell viability. (a) Cell viability of sarcoma cells treated with NAC after blue light (BL) irradiation for 48 h. PI, propidium iodide. (b, c) Flow cytometry analysis of apoptosis in sarcoma cells treated with NAC after BL irradiation for 48 h. UR, late apoptosis; LR, early apoptosis; UL, necrosis; LL, viable cells. (d) Western blotting analysis of the expression levels of apoptosis‐related proteins after BL irradiation in sarcoma cells treated with NAC. (e) Quantitative analysis of (d). Data are expressed as the mean ± standard error of the mean of at least three independent experiments. *p < 0.05, **p < 0.01, ***p < 0.001, ****p < 0.0001.

As shown in Figure 2, ROS‐based cell injury occurs in sarcoma cells in response to blue LED irradiation. We investigated whether N‐acetylcysteine (NAC), a ROS scavenger, could restore the sarcoma cell growth inhibition induced by blue LED irradiation. As shown in Figure S11, NAC reduced ROS levels in sarcoma cells induced by blue LED irradiation. A CCK8 assay showed that NAC treatment alleviated the decrease in cell viability in all sarcoma cell lines (Figure 6a). An apoptosis detection assay using Annexin V/propidium iodide revealed that NAC treatment of sarcoma cells reduced the numbers of apoptotic cells generated by blue LED in all sarcoma cell lines (Figure 6b,c). Furthermore, western blotting showed that the expression of cleaved PARP was significantly attenuated in NAC‐treated sarcoma cells compared with the blue LED‐irradiated group (Figure 6d,e and S12). These results suggested that blue LED irradiation induced ROS‐dependent apoptosis and that this apoptosis reduced the viability of sarcoma cells exposed to blue LED.

### 3.7 ROS From Blue LED Irradiation Causes Mitochondrial Damage and Autophagy in Sarcoma Cells

**FIGURE 7:**
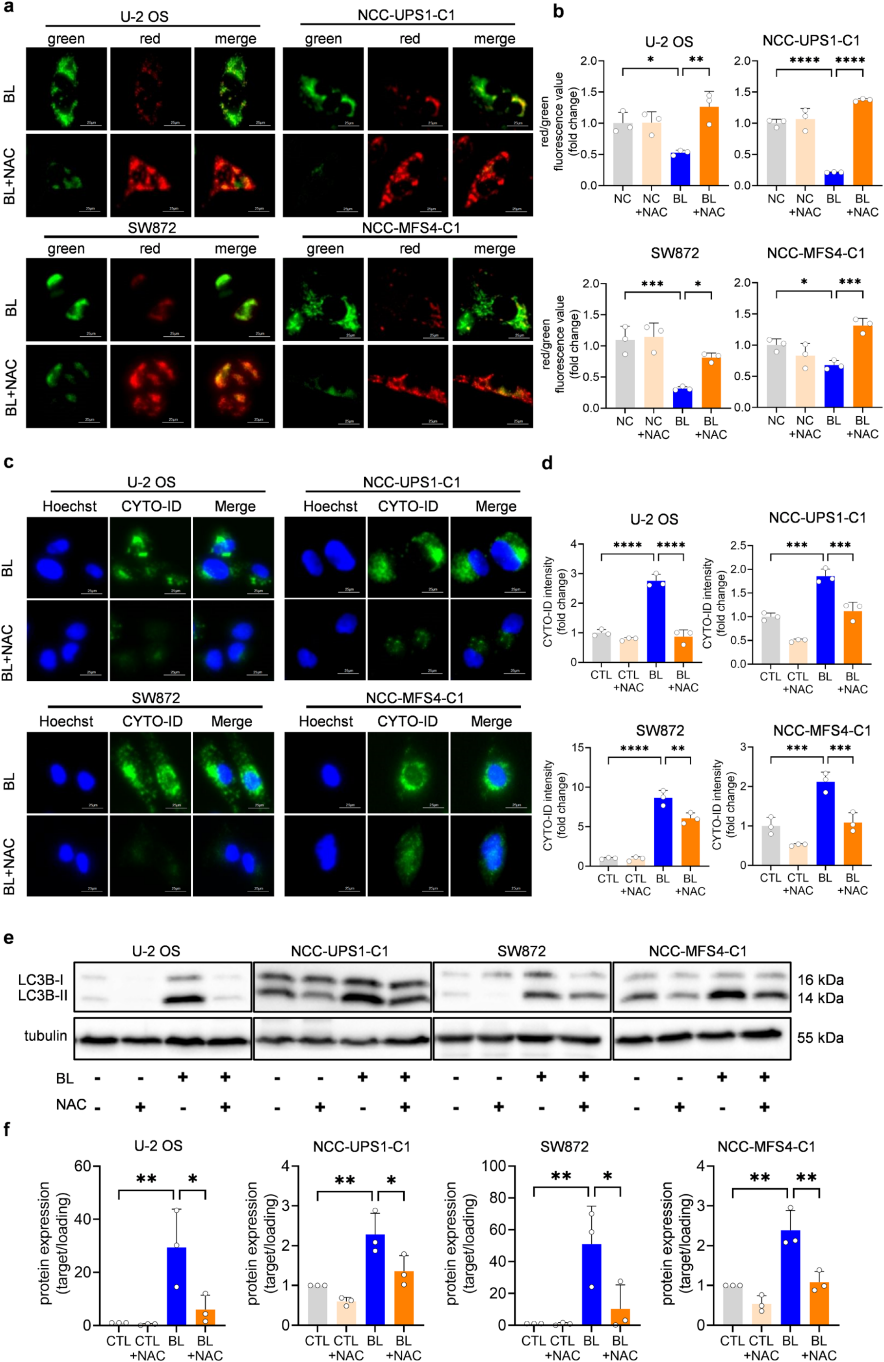
Blue light‐derived reactive oxygen species induce mitochondrial dysfunction and autophagy in sarcoma cells. N‐Acetyl‐cysteine (NAC) suppresses blue light‐derived mitochondrial dysfunction and autophagy. (a, b) Representative fluorescence images and quantitative analysis of the mitochondrial membrane potential using a JC‐I assay in sarcoma cells treated with NAC after BL irradiation for 48 h. Scale bar = 25 μm. (c) Representative fluorescence images of autophagy using the CYTO‐ID assay in sarcoma cells treated with NAC after 48‐h irradiation. Scale bar = 25 μm. (d) Quantitative analysis of (c). (e) Western blotting analysis of the expression levels of autophagy‐related proteins after BL irradiation in NAC‐treated cells. (f) Quantitative analysis of (e). Data are expressed as the mean ± standard error of the mean of three independent experiments. *p < 0.05, **p < 0.01, ***p < 0.001, ****p < 0.0001.

Because we showed that blue LED irradiation induced mitochondrial damage and autophagy in sarcoma cells (Figures 4 and 5), we studied whether these phenomena were caused by ROS derived from blue LED irradiation. First, the ΔΨm was evaluated using fluorescence. As shown in Figure 7a and Figure S13, under a fluorescence microscope, JC‐1 aggregates (red) in NAC‐treated sarcoma cells were increased with blue LED irradiation compared with the non‐NAC‐treated group. Flow cytometry also indicated a significant elevation of red fluorescence in the NAC‐treated BL irradiation group in all sarcoma cell lines (Figure 7b). The results suggested that NAC alleviated the decrease in the mitochondrial membrane potential, that is, mitochondrial function. In a CYTO‐ID autophagy detection assay, both fluorescence microscopy and flow cytometry revealed that the green fluorescence from blue LED irradiation was attenuated in NAC‐treated sarcoma cell lines (Figures 7c,d and S14). In addition, western blotting showed that the expression of LC3B‐II in the NAC‐treated group was significantly attenuated compared to that in the group irradiated with blue LED alone (Figure 7e,f and S15). These results indicated that ROS generated by blue LED irradiation induced autophagy in sarcoma cells.

### 3.8 In Undifferentiated Pleomorphic Sarcoma Cells, Blue LED Light Induces Apoptosis via an Endogenous Pathway Mediated by Mitochondria, and Autophagy Inhibits the Apoptosis

**FIGURE 8:**
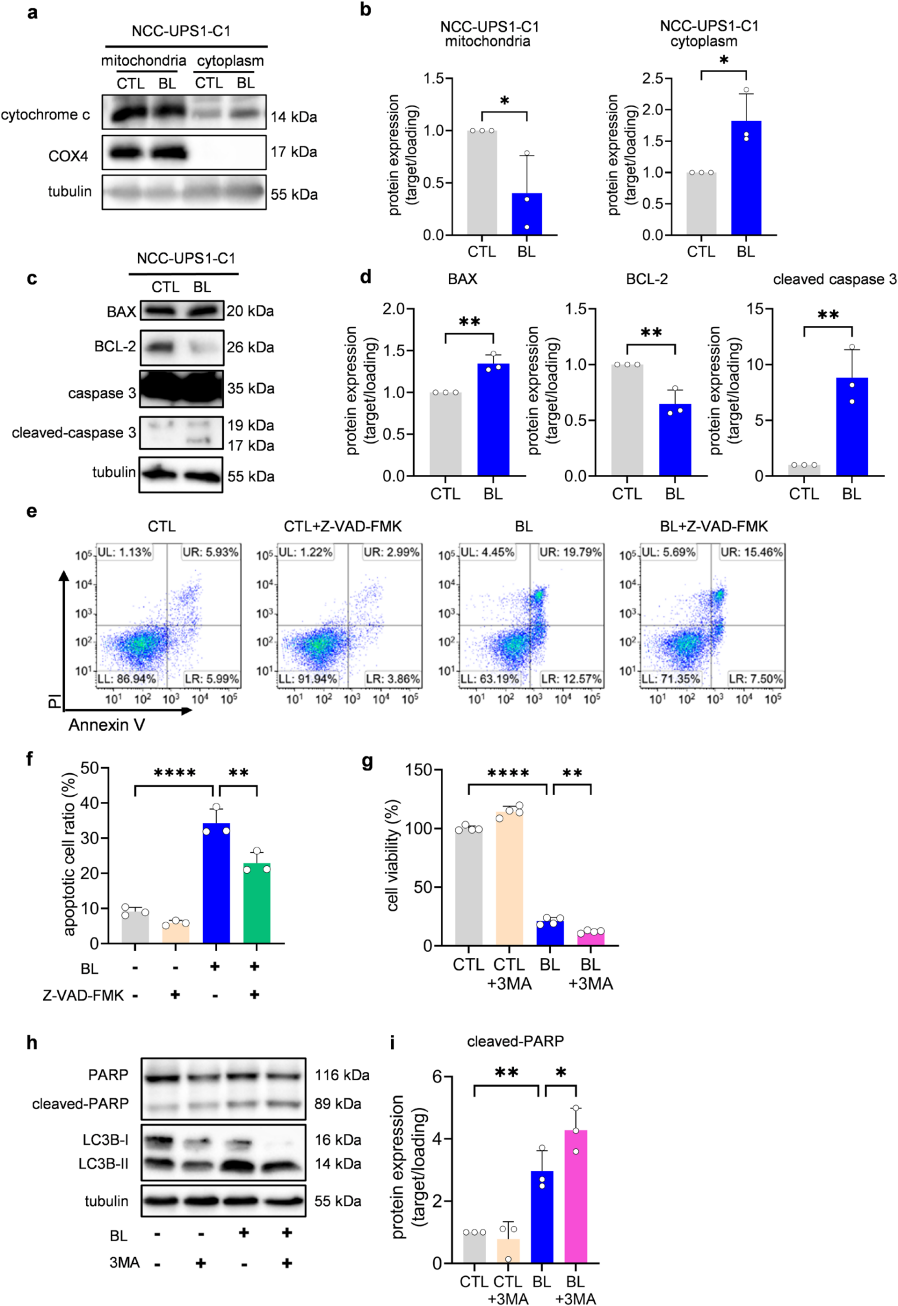
Blue light activates endogenous apoptosis pathways via the BCL‐2 family in NCC‐UPS1‐C1 cells. Blue light‐induced autophagy acts against apoptosis in NCC‐UPS1‐C1 cells. (a) Western blotting analysis of the expression level of cytochrome c in the mitochondria and cytoplasm of UPS cells after blue light (BL) irradiation. (b) Quantitative analysis of (a). (c) Western blotting analysis of the protein expression levels of the BCL‐2 family and caspase pathway in UPS cells after BL irradiation for 48 h. (d) Quantitative analysis of (c). (e, f) Flow cytometry analysis of apoptosis in UPS cells treated with BL and the caspase inhibitor Z‐VAD‐FMK. PI, propidium iodide. UR, late apoptosis; LR, early apoptosis; UL, necrosis; LL, viable cells. (g) Cell viability of UPS cells treated with the autophagy inhibitor 3‐methyladenine (3MA) after 48‐h BL irradiation. (h) Western blotting analysis of the expression levels of apoptosis and autophagy‐related proteins in UPS cells treated with BL and 3MA. (i) Quantitative analysis of (h). Data are presented as the mean ± standard error of the mean of three independent experiments. *p < 0.05, **p < 0.01, ****p < 0.0001.

As mentioned above, blue LED caused ROS‐mediated apoptosis, mitochondrial damage, and autophagy in sarcoma cells. However, it remained unclear how these processes were connected. We investigated the interrelationship of these phenomena in an undifferentiated pleomorphic sarcoma (UPS) cell line (NCC‐UPS1‐C1). Exposure of NCC‐UPS1‐C1 cells to blue LED significantly attenuated the expression of cytochrome c in mitochondria and significantly increased its expression in the cytoplasm compared with the non‐irradiated NCC‐UPS1‐C1 cells (Figures 8a,b and S16). This suggested that mitochondria were disrupted and that cytochrome c was released from the mitochondria. The expression of BAX, a promoter of apoptosis [18], was increased while that of the BCL‐2 negative regulator of apoptosis [18] was attenuated in the blue LED‐exposed group compared with the non‐irradiated group; moreover, the expression of cleaved caspase 3, which is located downstream of the endogenous apoptosis pathway, was markedly elevated (Figures 8c,d and S17). Furthermore, in UPS cells treated with Z‐VAD‐FMK (50 μm), a total caspase inhibitor, blue LED irradiation reduced the number of apoptotic cells compared with the non‐treated UPS cells (Figure 8e,f). These results suggested that blue LED irradiation induced endogenous apoptosis, in which cytochrome c in mitochondria was released into the cytoplasm via the BCL‐2 family and activated the caspase pathway.

Next, we investigated the relationship between apoptosis and autophagy. UPS cells were treated with the autophagy inhibitor 3‐methyladenine (3MA) and irradiated with blue LED to confirm the role of autophagy. As shown in Figure 8g, blue LED irradiation of 3MA‐treated UPS cells further reduced cell viability in the CCK8 assay. Furthermore, in western blotting, 3MA treatment increased the expression of cleaved PARP but attenuated that of the autophagy marker LC3B‐II (Figure 8h,i and S18). Taken together, autophagy produced by blue LED irradiation acted against apoptosis in UPS cells and protected them from blue LED irradiation, similar to our previous results for synovial sarcoma [11].

## 4 Discussion

The primary treatment for bone and soft tissue sarcomas of the extremities and trunk is wide resection, which would include normal tissues such as bone and muscle. Combined with adjuvant chemotherapy and radiation therapy, a good prognosis may be achieved. However, the wide resection often results in decreased postoperative ADLs due to the removal of healthy tissues as the margin, and most sarcomas are resistant to chemotherapy and radiotherapy, despite their side effects [19, 20]. Therefore, a treatment with high efficiency, minimally invasive properties, and fewer adverse effects is needed [21]. The present study demonstrated that blue LED light induced antitumor effects in bone and soft tissue sarcoma cell lines, as evidenced by inhibition of apoptosis‐mediated cell growth. This is the first such report for UPS, liposarcoma, and myxofibrosarcoma. Meanwhile, we found that LED light at 470 nm was minimally toxic to normal human skin fibroblasts. This study might provide a new therapeutic strategy that is cytotoxic to human sarcoma cells without injuring normal cells.

In this study, NHDFs, a human dermal fibroblast cell line, were used as normal cells for comparison with sarcoma cells because we assumed that the LED light would be used in actual clinical practice to irradiate sarcomas from the body surface. We found clear differences in intracellular responses to blue LED light between sarcoma cells and dermal fibroblasts. Although there have been many reports on the tumor growth inhibitory effects of blue LED light in various tumor cells [5, 6, 7, 8, 9, 10, 11], the responses of normal cells to BL irradiation have rarely been discussed. Regarding the effects of blue LED light on normal cells, Nakashima et al. reported that UVA produced ROS in the cytoplasm and nucleus of human keratinocyte cells, unlike BL, and concluded that short‐term exposure to BL does not result in a loss of cell viability in keratinocytes [22]. Hockberger et al. found that BL with an irradiance of 6.3 W/cm^2^ was cytotoxic in mouse fibroblast cells, monkey kidney epithelial cells, and human foreskin keratinocytes [23]; their BL was about 5000 times more intense than the blue LED light that we used (1.3 mW/cm^2^). Liebmann et al. mentioned that 453 nm BL did not cause keratinocyte toxicity at an irradiance of 68 mW/cm^2^ and a total irradiation energy of 500 J/cm^2^ or less [24], suggesting that the blue LED light used here may not cause cytotoxicity in normal cells due to its low irradiance. Furthermore, Opländer et al. showed toxic effects at 410 and 420 nm wavelengths but found that BL at 453 nm had almost no effect on cultured human dermal fibroblasts [25].

Based on these findings, BL in the 450–470 nm wavelength range has antitumor activity against malignant tumors and is not toxic to normal cells. We selected 470 nm BL based on a report that BL at 470 nm inhibited the growth of colorectal cancer [6]. We have previously shown the effect of BL at 470 nm on synovial sarcoma [11], and we used a similar wavelength in these experiments. We chose continuous BL irradiation at 1.3 mW/cm^2^ for 48 h because the cell viability assay in these experiments showed that continuous irradiation at 1.3 mW/cm^2^ for 48 h reduced the cell viability of all sarcoma cell lines by approximately 50%. Lockwood et al. reported differences in the response to blue LED light in normal epithelial keratinocyte cells and oral epithelial carcinoma, arguing that the amount of ROS and the way of ROS scavenging in each cell type are different [26]. Cells produce numerous oxidative scavengers, including HO‐1, to repair the damage caused by ROS [27]. HO‐1 catalyzes the first rate‐limiting step in the degradation of cellular heme and is one of the key components produced by the cell in response to oxidative stress [28]. In the present study, the expression of HO‐1 in NHDFs was elevated even though ROS was not detected in an ROS detection assay, suggesting that ROS was also generated in normal cells but eliminated by the oxidative stress‐processing mechanism in normal cells. This may be due to the difference in the amount of ROS and ROS processing mechanisms between malignant tumors and normal cells, as previously reported [26]. Therefore, BL with a low irradiance of 470 nm might specifically injure tumor cells without damaging normal tissues, especially normal skin. However, it has been reported that retinal cells, which are also normal cells, are sensitive to BL [29]. Accordingly, the response to blue LED light depends on the normal cell type, as well as whether it is a malignant tumor. The detailed processing mechanism of blue LED light‐induced ROS is unknown and should be investigated in a variety of normal cells, including normal rhabdomyocytes and adipocytes, as well as in human normal skin cells for medical application.

ROS produced by blue LED light is considered to inhibit the cell growth of malignant tumor cells [30]. In the present study, BL also induced ROS in bone and soft tissue sarcoma cells, and the elimination of ROS by using NAC increased cell viability, indicating that ROS induced by 470‐nm BL is the key to the inhibition of cell proliferation. However, controversy remains as to whether this occurs due to ROS‐dependent apoptotic cell death or ROS‐dependent autophagic cell death. Apoptosis is an important cellular event that involves a network of metabolic events activated by a variety of biological and physical stimuli. This process is characterized by the selective proteolysis of cytoplasmic and nuclear substrates that cannot maintain cellular homeostasis and is mediated by morphological changes and structural degradation [31, 32]. Teng et al. reported that irradiation of hepatocellular carcinoma cells with BL at 453 nm at 68 mW/cm^2^ caused mitochondrial membrane depolarization, resulting in apoptosis mediated by the BCL‐2 family [33]. We also previously reported that ROS‐dependent inhibition of cell proliferation by apoptosis occurred in synovial sarcoma, similar to Teng et al. [11]. In the present study, we evaluated the antitumor effects of blue LED light in several bone and soft tissue sarcoma cell lines and found that ROS‐dependent apoptosis occurred in all sarcoma cells as well. However, there are several reports of ROS‐dependent autophagic cell death [10]. Autophagy plays an important role in a variety of cellular processes in various cardiovascular diseases, neurodegenerative diseases, and cancers, although its role and mechanism are still unknown. He et al. reported that irradiation of osteosarcoma cell lines with 470 nm BL at 100 mW/cm^2^ caused cell growth inhibition through both ROS‐ and EGFR/Beclin‐1‐mediated autophagy signaling pathways [10]. In the present study, treatment with the autophagy inhibitor 3MA did not suppress cell death in UPS cells, but rather reduced cell viability and increased the expression of cleaved PARP, suggesting that autophagy derived from 470‐nm light did not induce cell death but did protect tumor cells from external stresses such as ROS. The response mechanisms of these cellular responses to BL may depend on the wavelength and illuminance of the irradiating light and on the cancer type, and further verification is needed.

The membrane potential of mitochondria that is formed by the impermeability of the inner mitochondrial membrane to protons is important for many mitochondrial functions, including ATP generation, protein import, replication, and fusion [34]. In this study, we found that ROS induced by blue LED light caused depolarization of mitochondrial membranes, resulting in mitochondrial dysfunction, including ATP production. Apoptotic stimuli, such as elevated levels of ROS, activate the BCL‐2 family death agonists BAX and BAK, which oligomerize to form an opening in the outer mitochondrial membrane. In addition, the mitochondrial permeability transition pore is formed. This large multimeric complex is thought to span both the outer and inner mitochondrial membranes. Then, cytochrome c passes through the mitochondrial membrane through these pores and is released into the cytoplasm [34]. The released cytochrome c binds to Apaf‐1 and forms apoptosomes, which activate the caspase pathway and lead to apoptosis [35]. In our previous report, blue LED‐associated ROS caused apoptosis of synovial sarcoma cells involving the caspase pathway, but it was not shown whether the cytochrome c/BCL‐2 family/caspase pathway, the intrinsic pathway of apoptosis, was activated by BL. The activation of this cytochrome c/BCL‐2 family/caspase pathway by BL was reported in only one study of the effect of BL on melanoma [7]. The present study showed that 470‐nm BL shifted cytochrome c localization in UPS cells from the mitochondria to the cytoplasm. As the mitochondrial membrane potential decreased, BCL‐2 family death agonists were activated, which increased the expression of cleaved caspase 3. In other words, oxidative stress, an apoptotic stimulus, triggers the release of cytochrome c from mitochondria and activates the intrinsic apoptosis pathway via the BCL‐2 family. This is the first report of the involvement of this pathway with BL in soft tissue sarcoma. Our results indicate that the intrinsic pathway of apoptosis occurs in mesenchymal malignant tumors as well as in epithelial malignant tumors such as melanoma [7].

In terms of the clinical applications of blue LED, external irradiation therapy is an option. The high irradiance of BL shown in previous reports [21] would be too toxic for normal cells such as skin for clinical applications. Low‐power blue LED light, as used in the present report, might be suitable for clinical application because it is less harmful to human skin. In our previous report on synovial sarcoma, BL irradiation did not cause adverse events in epidermal cells in vivo [11]. Therefore, our BL irradiation does not cause toxicity in normal tissues.

Another issue for the clinical application of BL is low tissue permeability. Photodynamic therapy applies a wavelength of 600–850 nm light in the actual clinical setting because of tissue permeability [36]. Although the application of external BL irradiation to nude mice showed an antitumor effect [8, 11], there is a possibility that the light will not penetrate human skin and that the light irradiance will be attenuated because the human skin permeability of 440 nm BL is about 1 mm [37, 38]. Therefore, new wireless LED devices that can be implanted closer to the tumor are needed. As far as we know, there are no commercially available wireless LED devices that can be implanted in vivo. It is therefore necessary to devise implantable LED systems that show antitumor effects.

## 5 Conclusion

We have reported on the antitumor effects of continuous low‐power irradiation of BL on various bone and soft tissue sarcomas and the mechanisms underlying these effects. The low‐power BL used in our experiments may exert antitumor effects specifically on the sarcomas without damaging normal cells.

## Author Contributions

**Shinji Kawaguchi:** conceptualization (equal), data curation (lead), formal analysis (lead), investigation (lead), methodology (equal), software (lead), validation (lead), visualization (lead), writing – original draft (lead). **Toshihiko Nishisho:** conceptualization (lead), project administration (lead), writing – review and editing (lead). **Shunichi Toki:** conceptualization (equal), project administration (equal), writing – review and editing (equal). **Makoto Takeuchi:** conceptualization (equal), data curation (equal), investigation (supporting), methodology (lead), software (equal), visualization (equal). **Shunsuke Tamaki:** data curation (equal), investigation (equal). **Koichi Sairyo:** conceptualization (equal), funding acquisition (lead), project administration (equal), resources (lead), supervision (lead).

## Ethics Statement

No human and animal studies were used in this study. We were given permission for the use of cell lines, NCC‐UPS1‐C1 (undifferentiated pleomorphic sarcoma) and NCC‐MFS4‐C1 (myxofibrosarcoma) from Dr. Tadashi Kondo (Division of Rare Cancer Research, National Cancer Center Research Institute, Japan), which was established at the National Cancer Center Research Institute, Japan.

## Conflicts of Interest

The authors declare no conflicts of interest.

## Supporting information


**Figure S1.** Figure of a multi‐well plate placed in the LED device.
**Figure S2.** The parameters of the LED light source used in these experiments. (a) Temperature of the culture medium in the plate with the LED device used. The LED light intensity used in this experiment (0.1, 0.6, and 1.3 mW/cm^2^) did not increase the temperature of the medium in a multi‐well plate nearly as much as in the non‐irradiated group (0 mW/cm^2^). (b) The light dose was calculated using the following formula: light dose (J/cm^2^) = light intensity (W/cm^2^) × irradiation time (seconds).
**Figure S3.** Cell morphology of U‐2 OS, NCC‐UPS1‐C1, SW872, NCC‐MFS4‐C1, and NHDF cells with or without blue light (1.3 mW/cm^2^) for 48 h.
**Figure S4.** Effects of blue LED irradiation on invasion in sarcoma cells. (a) Wound healing assay of sarcoma cells with continuous blue LED irradiation. Scale bar = 100 μm. (b) Quantification of the mean percentage of wound distance in each group. *p < 0.05, **p < 0.01, ***p < 0.001.
**Figure S5.** Effects of blue LED irradiation on migration in sarcoma cells. (a) Migration abilities, as measured using Transwell filters. (b) Quantification of the mean number of migrated cells in each group. Data are expressed as the mean ± standard error of the mean of three independent experiments. **p < 0.01, ****p < 0.0001.
**Figure S6.** Original uncropped western blots from Figure 2e.
**Figure S7.** The sarcoma cells and the normal dermal cells were irradiated with blue light (1.3 mW/cm^2^) for 48 h, and then mRNA expression of HO‐1 and oxidative stress induced growth inhibitor 1 (OSGIN1) was measured by qPCR. Data are expressed as the mean ± standard error of the mean of at least three independent experiments. *p < 0.05, **p < 0.01, ***p < 0.001.
**Figure S8.** Density plots of flow cytometry analysis of apoptosis in NHDF cells after blue light irradiation with staurosporine treatment for 24 h as positive control.
**Figure S9.** Original uncropped western blots from Figure 3c.
**Figure S10.** Original uncropped western blots from Figure 5c.
**Figure S11.** Quantitative analysis of intracellular ROS detection with the CellROX assay in sarcoma cells treated with NAC.
**Figure S12.** Original uncropped western blots from Figure 6d.
**Figure S13.** Fluorescence images of the JC‐1 assay in Figure 7a including CTL and CTL + NAC.
**Figure S14.** Fluorescence images of the CYTO‐ID assay in Figure 7c including CTL and CTL + NAC.
**Figure S15.** Original uncropped western blots from Figure 7e.
**Figure S16.** Original uncropped western blots from Figure 8a.
**Figure S17.** Original uncropped western blots from Figure 8c.
**Figure S18.** Original uncropped western blots from Figure 8h.

**Table S1.** Reagents used in this study.
**Table S2.** Sequence of primers used for qPCR.

## Data Availability

The data used in this study are available from the corresponding author upon reasonable request.
