# Supplementary figures and images for: Blue Light Emitting Diode Suppresses Sarcoma Cell Proliferation via the Endogenous Apoptotic Pathway Without Damaging Normal Cells
Source: Cancer Med. 2025 Mar 24;14(6):e70770. doi: 10.1002/cam4.70770 (PMC11931449; doi:10.1002/cam4.70770)

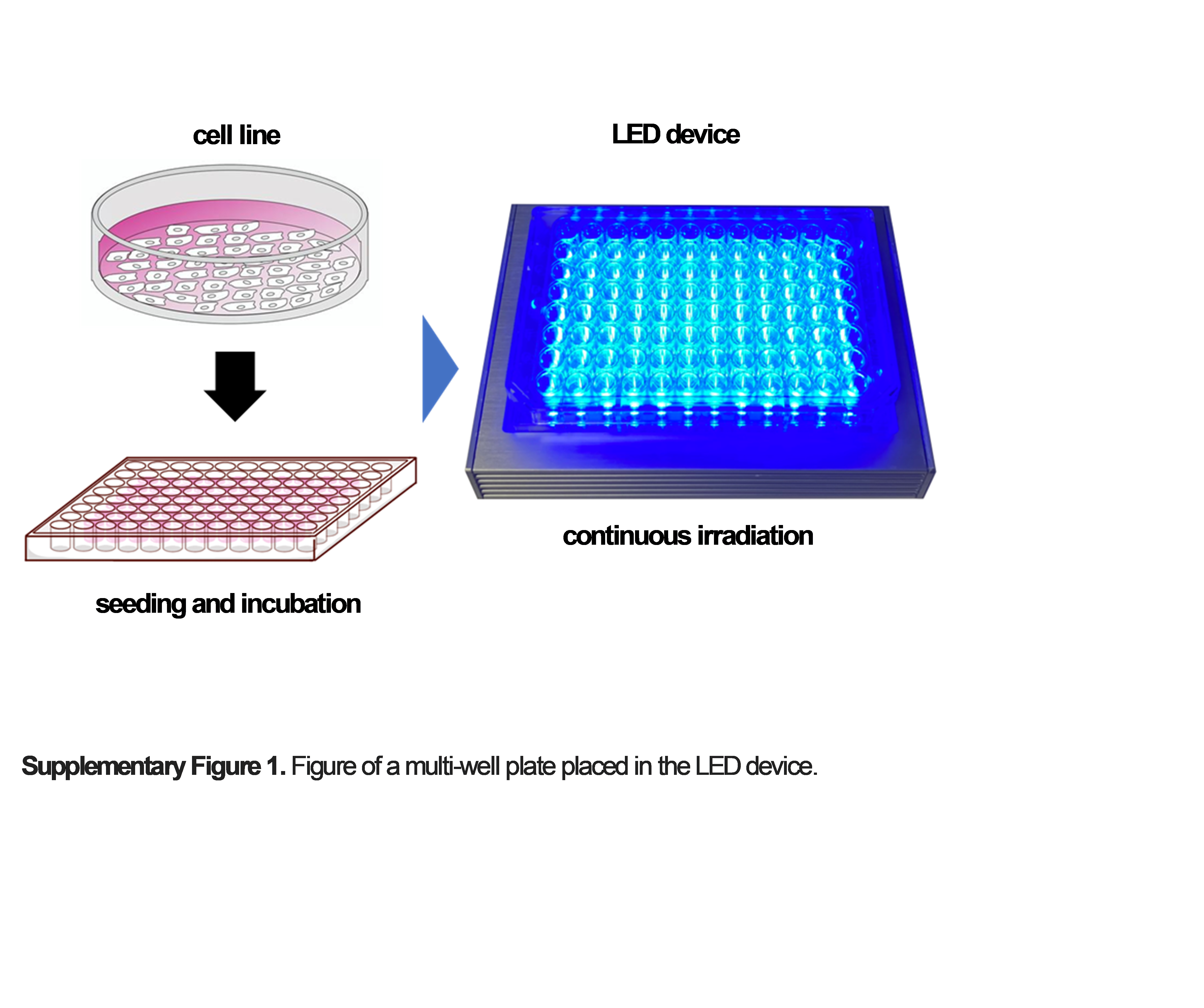

Supplement: Supplementary file 1 — Figure S1. Figure of a multi‐well plate placed in the LED device. Figure S2. The parameters of the LED light source used in these experiments. (a) Temperature of the culture medium in the plate with the LED device used. The LED light intensity used in this experiment (0.1, 0.6, and 1.3 mW/cm2) did not increase the temperature of the medium in a multi‐well plate nearly as much as in the non‐irradiated group (0 mW/cm2). (b) The light dose was calculated using the following formula: light dose (J/cm2) = light intensity (W/cm2) × irradiation time (seconds). Figure S3. Cell morphology of U‐2 OS, NCC‐UPS1‐C1, SW872, NCC‐MFS4‐C1, and NHDF cells with or without blue light (1.3 mW/cm2) for 48 h. Figure S4. Effects of blue LED irradiation on invasion in sarcoma cells. (a) Wound healing assay of sarcoma cells with continuous blue LED irradiation. Scale bar = 100 μm. (b) Quantification of the mean percentage of wound distance in each group. *p < 0.05, **p < 0.01, ***p < 0.001. Figure S5. Effects of blue LED irradiation on migration in sarcoma cells. (a) Migration abilities, as measured using Transwell filters. (b) Quantification of the mean number of migrated cells in each group. Data are expressed as the mean ± standard error of the mean of three independent experiments. **p < 0.01, ****p < 0.0001. Figure S6. Original uncropped western blots from Figure 2e. Figure S7. The sarcoma cells and the normal dermal cells were irradiated with blue light (1.3 mW/cm2) for 48 h, and then mRNA expression of HO‐1 and oxidative stress induced growth inhibitor 1 (OSGIN1) was measured by qPCR. Data are expressed as the mean ± standard error of the mean of at least three independent experiments. *p < 0.05, **p < 0.01, ***p < 0.001. Figure S8. Density plots of flow cytometry analysis of apoptosis in NHDF cells after blue light irradiation with staurosporine treatment for 24 h as positive control. Figure S9. Original uncropped western blots from Figure 3c. Figure S10. Original uncropped western b [file CAM4-14-e70770-s002.zip › cam470770-sup-0001-supinfo_supplementary figure1-1.tif]

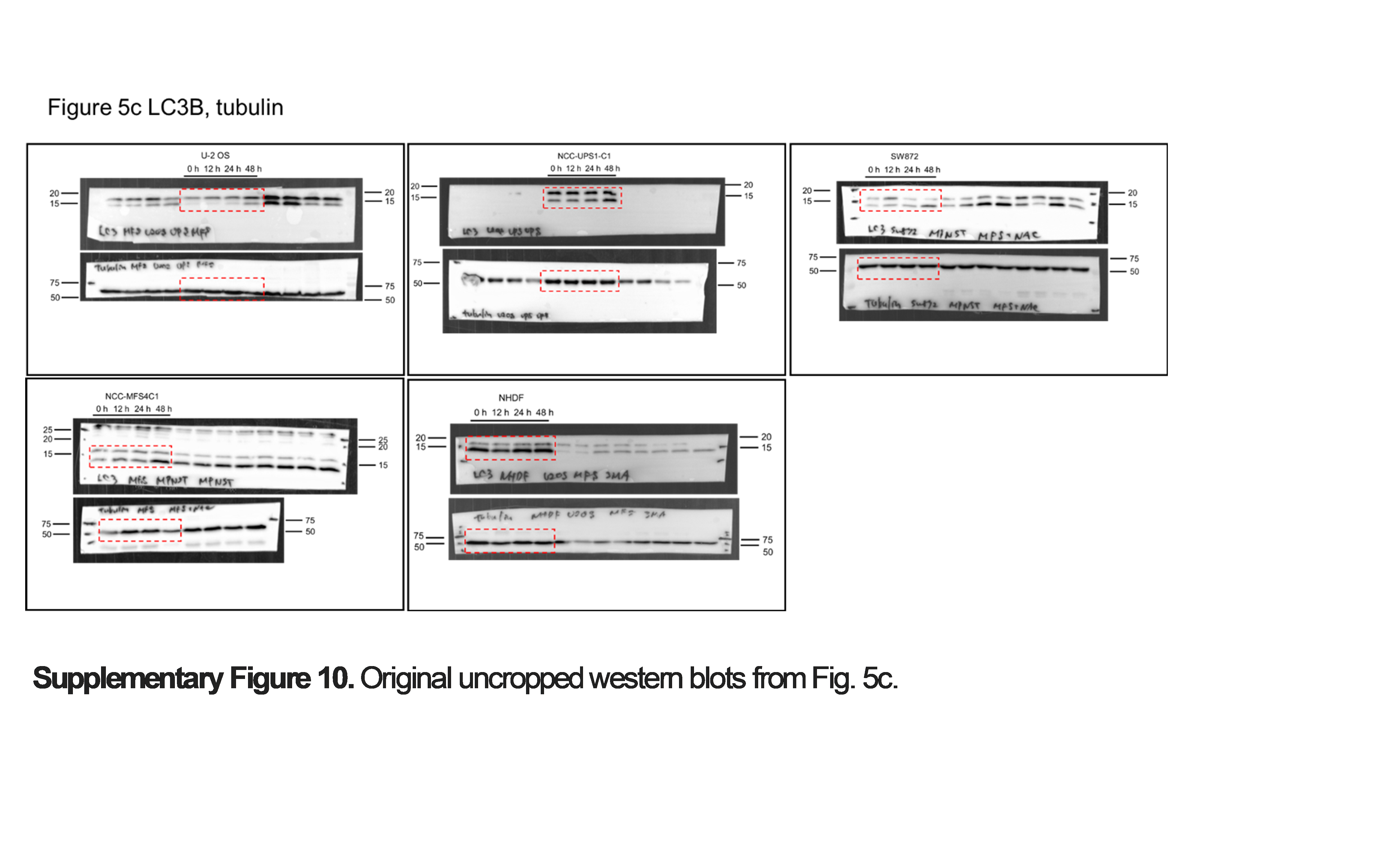

Supplement: Supplementary file 1 — Figure S1. Figure of a multi‐well plate placed in the LED device. Figure S2. The parameters of the LED light source used in these experiments. (a) Temperature of the culture medium in the plate with the LED device used. The LED light intensity used in this experiment (0.1, 0.6, and 1.3 mW/cm2) did not increase the temperature of the medium in a multi‐well plate nearly as much as in the non‐irradiated group (0 mW/cm2). (b) The light dose was calculated using the following formula: light dose (J/cm2) = light intensity (W/cm2) × irradiation time (seconds). Figure S3. Cell morphology of U‐2 OS, NCC‐UPS1‐C1, SW872, NCC‐MFS4‐C1, and NHDF cells with or without blue light (1.3 mW/cm2) for 48 h. Figure S4. Effects of blue LED irradiation on invasion in sarcoma cells. (a) Wound healing assay of sarcoma cells with continuous blue LED irradiation. Scale bar = 100 μm. (b) Quantification of the mean percentage of wound distance in each group. *p < 0.05, **p < 0.01, ***p < 0.001. Figure S5. Effects of blue LED irradiation on migration in sarcoma cells. (a) Migration abilities, as measured using Transwell filters. (b) Quantification of the mean number of migrated cells in each group. Data are expressed as the mean ± standard error of the mean of three independent experiments. **p < 0.01, ****p < 0.0001. Figure S6. Original uncropped western blots from Figure 2e. Figure S7. The sarcoma cells and the normal dermal cells were irradiated with blue light (1.3 mW/cm2) for 48 h, and then mRNA expression of HO‐1 and oxidative stress induced growth inhibitor 1 (OSGIN1) was measured by qPCR. Data are expressed as the mean ± standard error of the mean of at least three independent experiments. *p < 0.05, **p < 0.01, ***p < 0.001. Figure S8. Density plots of flow cytometry analysis of apoptosis in NHDF cells after blue light irradiation with staurosporine treatment for 24 h as positive control. Figure S9. Original uncropped western blots from Figure 3c. Figure S10. Original uncropped western b [file CAM4-14-e70770-s002.zip › cam470770-sup-0002-supinfo_supplementary figure10-1.tif]

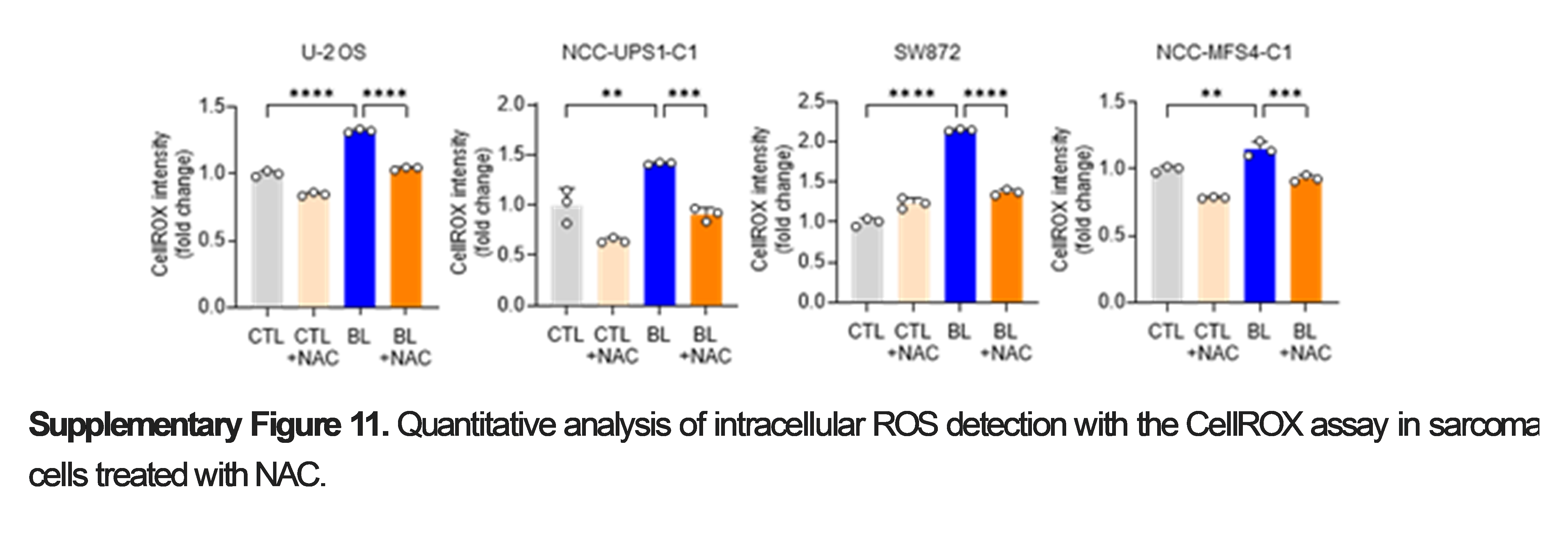

Supplement: Supplementary file 1 — Figure S1. Figure of a multi‐well plate placed in the LED device. Figure S2. The parameters of the LED light source used in these experiments. (a) Temperature of the culture medium in the plate with the LED device used. The LED light intensity used in this experiment (0.1, 0.6, and 1.3 mW/cm2) did not increase the temperature of the medium in a multi‐well plate nearly as much as in the non‐irradiated group (0 mW/cm2). (b) The light dose was calculated using the following formula: light dose (J/cm2) = light intensity (W/cm2) × irradiation time (seconds). Figure S3. Cell morphology of U‐2 OS, NCC‐UPS1‐C1, SW872, NCC‐MFS4‐C1, and NHDF cells with or without blue light (1.3 mW/cm2) for 48 h. Figure S4. Effects of blue LED irradiation on invasion in sarcoma cells. (a) Wound healing assay of sarcoma cells with continuous blue LED irradiation. Scale bar = 100 μm. (b) Quantification of the mean percentage of wound distance in each group. *p < 0.05, **p < 0.01, ***p < 0.001. Figure S5. Effects of blue LED irradiation on migration in sarcoma cells. (a) Migration abilities, as measured using Transwell filters. (b) Quantification of the mean number of migrated cells in each group. Data are expressed as the mean ± standard error of the mean of three independent experiments. **p < 0.01, ****p < 0.0001. Figure S6. Original uncropped western blots from Figure 2e. Figure S7. The sarcoma cells and the normal dermal cells were irradiated with blue light (1.3 mW/cm2) for 48 h, and then mRNA expression of HO‐1 and oxidative stress induced growth inhibitor 1 (OSGIN1) was measured by qPCR. Data are expressed as the mean ± standard error of the mean of at least three independent experiments. *p < 0.05, **p < 0.01, ***p < 0.001. Figure S8. Density plots of flow cytometry analysis of apoptosis in NHDF cells after blue light irradiation with staurosporine treatment for 24 h as positive control. Figure S9. Original uncropped western blots from Figure 3c. Figure S10. Original uncropped western b [file CAM4-14-e70770-s002.zip › cam470770-sup-0003-supinfo_supplementary figure11-1.tif]

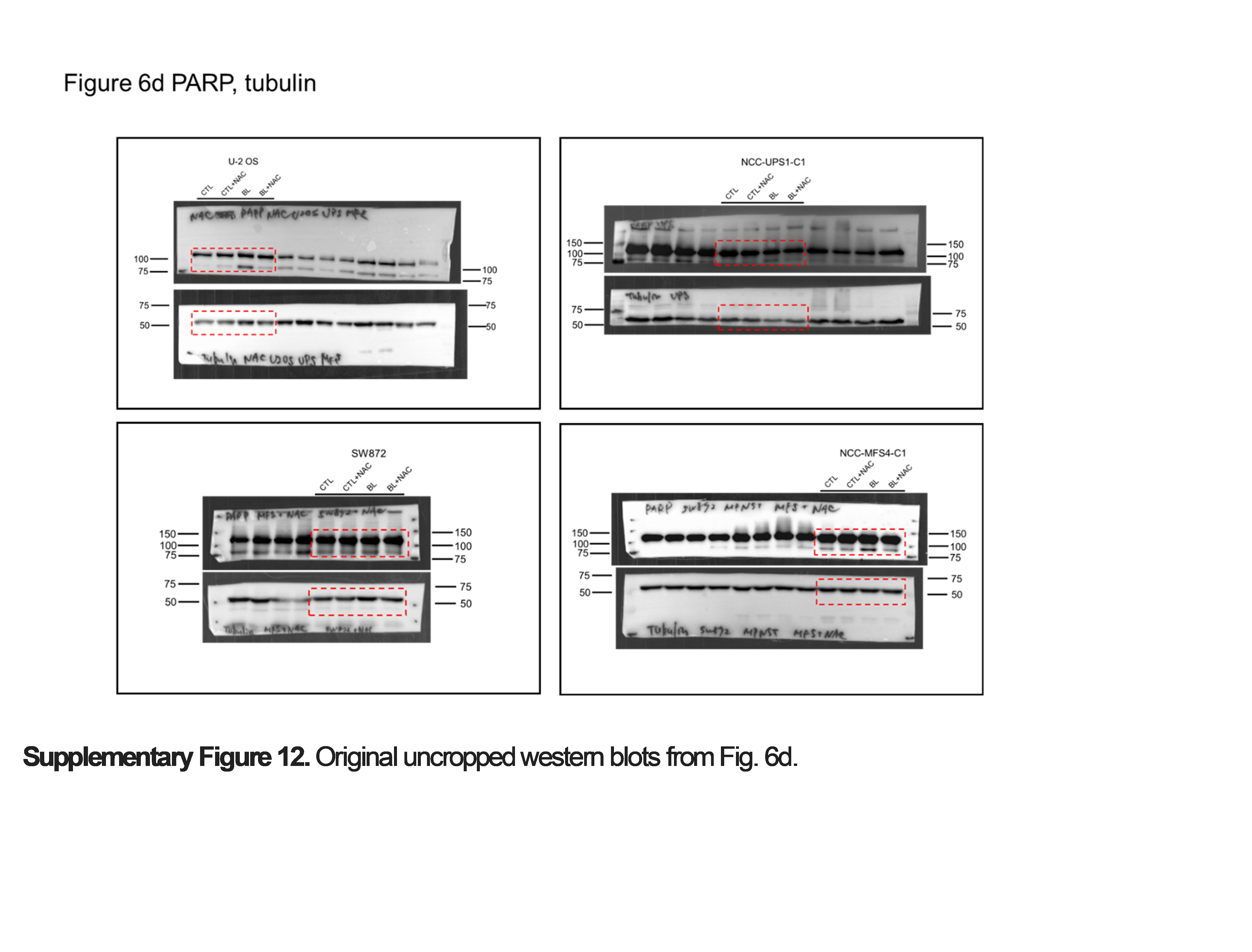

Supplement: Supplementary file 1 — Figure S1. Figure of a multi‐well plate placed in the LED device. Figure S2. The parameters of the LED light source used in these experiments. (a) Temperature of the culture medium in the plate with the LED device used. The LED light intensity used in this experiment (0.1, 0.6, and 1.3 mW/cm2) did not increase the temperature of the medium in a multi‐well plate nearly as much as in the non‐irradiated group (0 mW/cm2). (b) The light dose was calculated using the following formula: light dose (J/cm2) = light intensity (W/cm2) × irradiation time (seconds). Figure S3. Cell morphology of U‐2 OS, NCC‐UPS1‐C1, SW872, NCC‐MFS4‐C1, and NHDF cells with or without blue light (1.3 mW/cm2) for 48 h. Figure S4. Effects of blue LED irradiation on invasion in sarcoma cells. (a) Wound healing assay of sarcoma cells with continuous blue LED irradiation. Scale bar = 100 μm. (b) Quantification of the mean percentage of wound distance in each group. *p < 0.05, **p < 0.01, ***p < 0.001. Figure S5. Effects of blue LED irradiation on migration in sarcoma cells. (a) Migration abilities, as measured using Transwell filters. (b) Quantification of the mean number of migrated cells in each group. Data are expressed as the mean ± standard error of the mean of three independent experiments. **p < 0.01, ****p < 0.0001. Figure S6. Original uncropped western blots from Figure 2e. Figure S7. The sarcoma cells and the normal dermal cells were irradiated with blue light (1.3 mW/cm2) for 48 h, and then mRNA expression of HO‐1 and oxidative stress induced growth inhibitor 1 (OSGIN1) was measured by qPCR. Data are expressed as the mean ± standard error of the mean of at least three independent experiments. *p < 0.05, **p < 0.01, ***p < 0.001. Figure S8. Density plots of flow cytometry analysis of apoptosis in NHDF cells after blue light irradiation with staurosporine treatment for 24 h as positive control. Figure S9. Original uncropped western blots from Figure 3c. Figure S10. Original uncropped western b [file CAM4-14-e70770-s002.zip › cam470770-sup-0004-supinfo_supplementary figure12-1.tif]

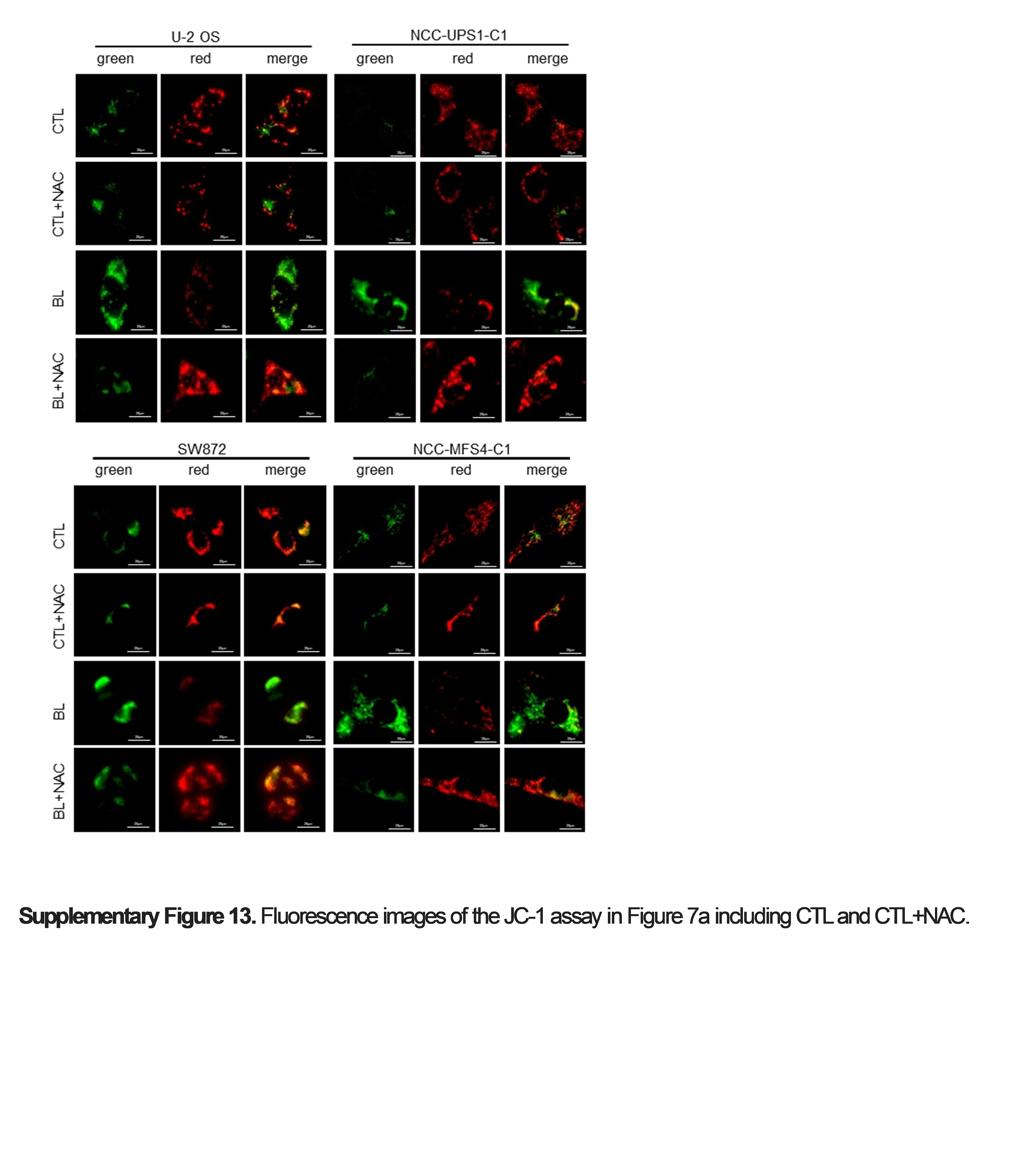

Supplement: Supplementary file 1 — Figure S1. Figure of a multi‐well plate placed in the LED device. Figure S2. The parameters of the LED light source used in these experiments. (a) Temperature of the culture medium in the plate with the LED device used. The LED light intensity used in this experiment (0.1, 0.6, and 1.3 mW/cm2) did not increase the temperature of the medium in a multi‐well plate nearly as much as in the non‐irradiated group (0 mW/cm2). (b) The light dose was calculated using the following formula: light dose (J/cm2) = light intensity (W/cm2) × irradiation time (seconds). Figure S3. Cell morphology of U‐2 OS, NCC‐UPS1‐C1, SW872, NCC‐MFS4‐C1, and NHDF cells with or without blue light (1.3 mW/cm2) for 48 h. Figure S4. Effects of blue LED irradiation on invasion in sarcoma cells. (a) Wound healing assay of sarcoma cells with continuous blue LED irradiation. Scale bar = 100 μm. (b) Quantification of the mean percentage of wound distance in each group. *p < 0.05, **p < 0.01, ***p < 0.001. Figure S5. Effects of blue LED irradiation on migration in sarcoma cells. (a) Migration abilities, as measured using Transwell filters. (b) Quantification of the mean number of migrated cells in each group. Data are expressed as the mean ± standard error of the mean of three independent experiments. **p < 0.01, ****p < 0.0001. Figure S6. Original uncropped western blots from Figure 2e. Figure S7. The sarcoma cells and the normal dermal cells were irradiated with blue light (1.3 mW/cm2) for 48 h, and then mRNA expression of HO‐1 and oxidative stress induced growth inhibitor 1 (OSGIN1) was measured by qPCR. Data are expressed as the mean ± standard error of the mean of at least three independent experiments. *p < 0.05, **p < 0.01, ***p < 0.001. Figure S8. Density plots of flow cytometry analysis of apoptosis in NHDF cells after blue light irradiation with staurosporine treatment for 24 h as positive control. Figure S9. Original uncropped western blots from Figure 3c. Figure S10. Original uncropped western b [file CAM4-14-e70770-s002.zip › cam470770-sup-0005-supinfo_supplementary figure13-1.tif]

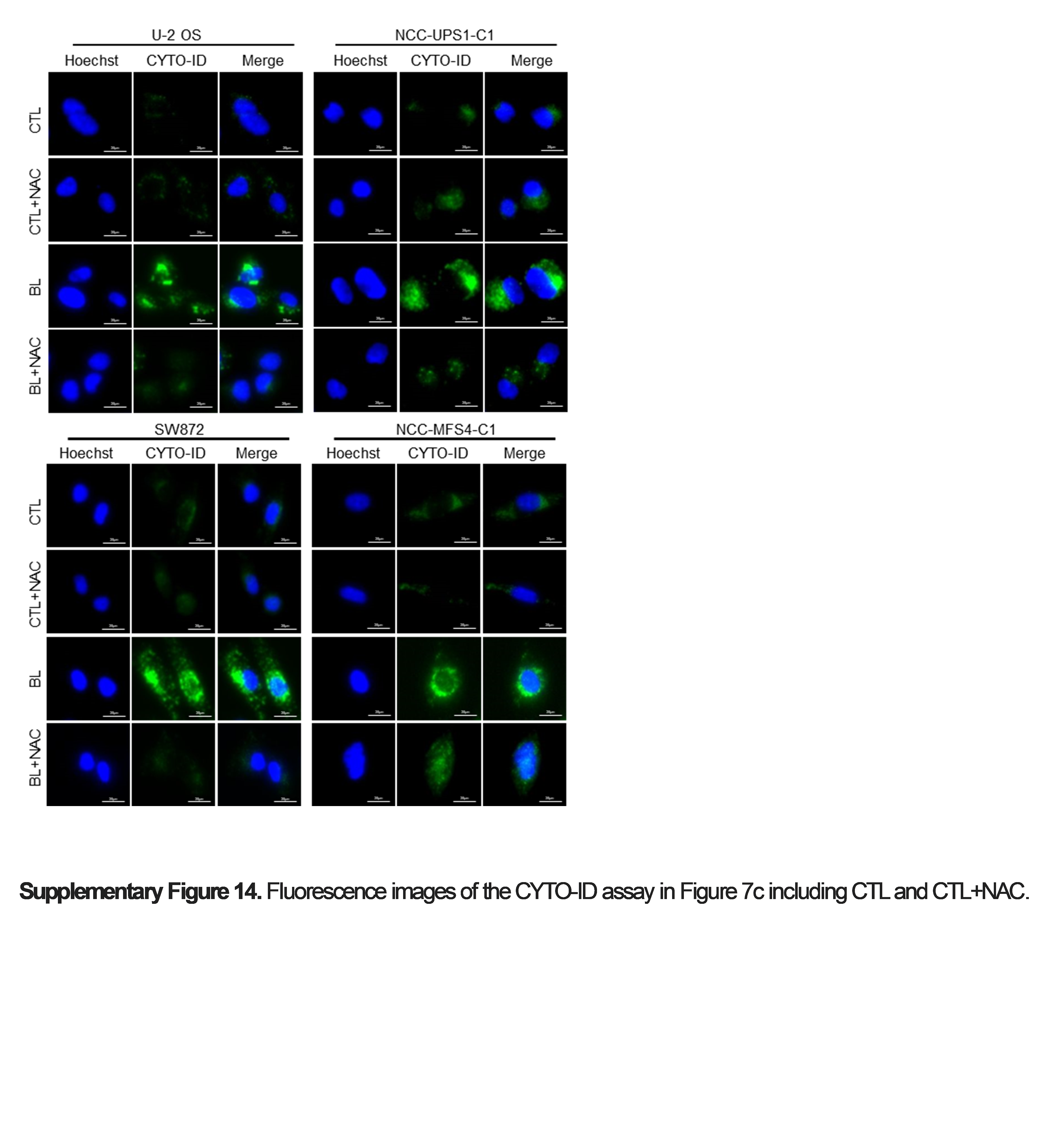

Supplement: Supplementary file 1 — Figure S1. Figure of a multi‐well plate placed in the LED device. Figure S2. The parameters of the LED light source used in these experiments. (a) Temperature of the culture medium in the plate with the LED device used. The LED light intensity used in this experiment (0.1, 0.6, and 1.3 mW/cm2) did not increase the temperature of the medium in a multi‐well plate nearly as much as in the non‐irradiated group (0 mW/cm2). (b) The light dose was calculated using the following formula: light dose (J/cm2) = light intensity (W/cm2) × irradiation time (seconds). Figure S3. Cell morphology of U‐2 OS, NCC‐UPS1‐C1, SW872, NCC‐MFS4‐C1, and NHDF cells with or without blue light (1.3 mW/cm2) for 48 h. Figure S4. Effects of blue LED irradiation on invasion in sarcoma cells. (a) Wound healing assay of sarcoma cells with continuous blue LED irradiation. Scale bar = 100 μm. (b) Quantification of the mean percentage of wound distance in each group. *p < 0.05, **p < 0.01, ***p < 0.001. Figure S5. Effects of blue LED irradiation on migration in sarcoma cells. (a) Migration abilities, as measured using Transwell filters. (b) Quantification of the mean number of migrated cells in each group. Data are expressed as the mean ± standard error of the mean of three independent experiments. **p < 0.01, ****p < 0.0001. Figure S6. Original uncropped western blots from Figure 2e. Figure S7. The sarcoma cells and the normal dermal cells were irradiated with blue light (1.3 mW/cm2) for 48 h, and then mRNA expression of HO‐1 and oxidative stress induced growth inhibitor 1 (OSGIN1) was measured by qPCR. Data are expressed as the mean ± standard error of the mean of at least three independent experiments. *p < 0.05, **p < 0.01, ***p < 0.001. Figure S8. Density plots of flow cytometry analysis of apoptosis in NHDF cells after blue light irradiation with staurosporine treatment for 24 h as positive control. Figure S9. Original uncropped western blots from Figure 3c. Figure S10. Original uncropped western b [file CAM4-14-e70770-s002.zip › cam470770-sup-0006-supinfo_supplementary figure14-1.tif]

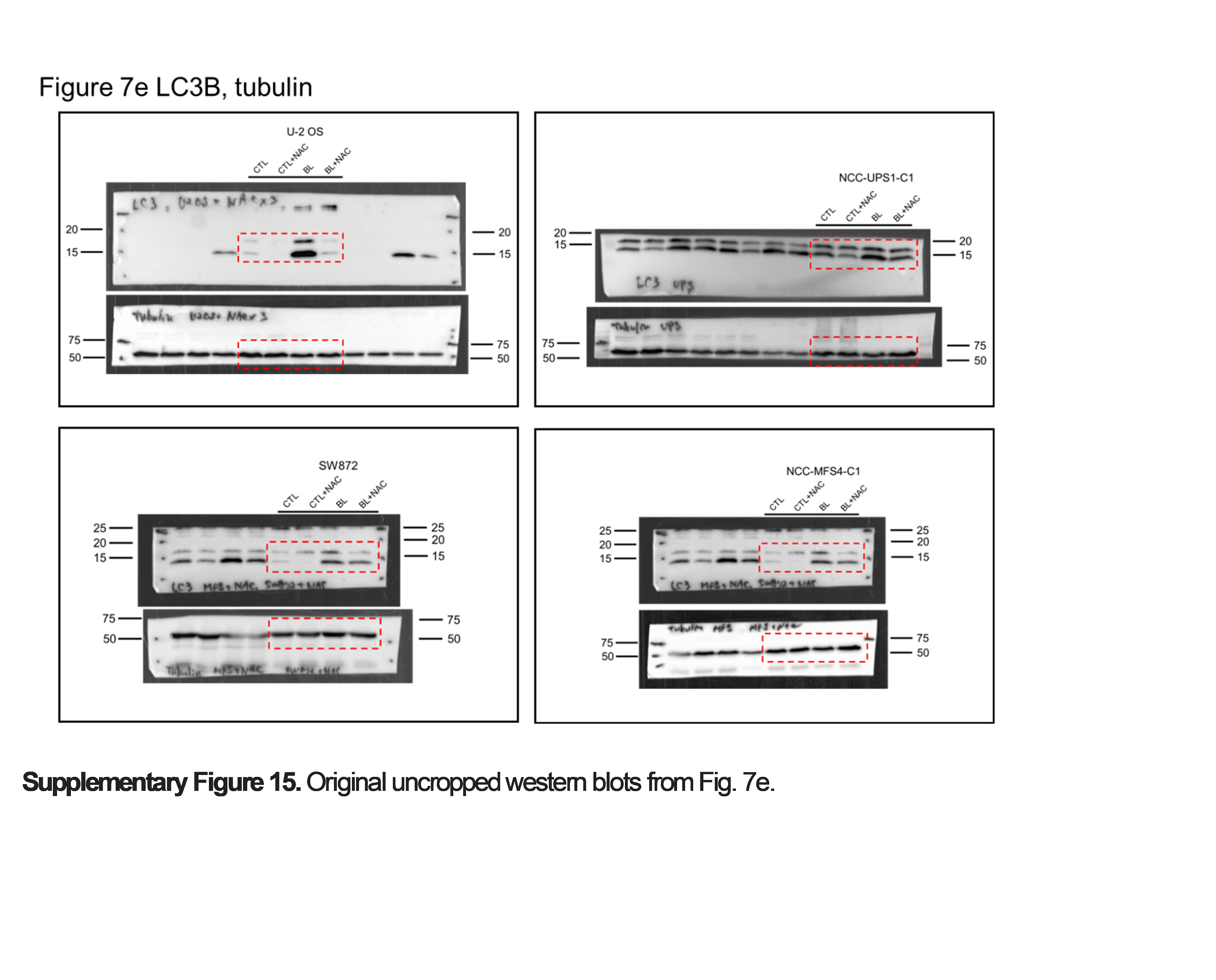

Supplement: Supplementary file 1 — Figure S1. Figure of a multi‐well plate placed in the LED device. Figure S2. The parameters of the LED light source used in these experiments. (a) Temperature of the culture medium in the plate with the LED device used. The LED light intensity used in this experiment (0.1, 0.6, and 1.3 mW/cm2) did not increase the temperature of the medium in a multi‐well plate nearly as much as in the non‐irradiated group (0 mW/cm2). (b) The light dose was calculated using the following formula: light dose (J/cm2) = light intensity (W/cm2) × irradiation time (seconds). Figure S3. Cell morphology of U‐2 OS, NCC‐UPS1‐C1, SW872, NCC‐MFS4‐C1, and NHDF cells with or without blue light (1.3 mW/cm2) for 48 h. Figure S4. Effects of blue LED irradiation on invasion in sarcoma cells. (a) Wound healing assay of sarcoma cells with continuous blue LED irradiation. Scale bar = 100 μm. (b) Quantification of the mean percentage of wound distance in each group. *p < 0.05, **p < 0.01, ***p < 0.001. Figure S5. Effects of blue LED irradiation on migration in sarcoma cells. (a) Migration abilities, as measured using Transwell filters. (b) Quantification of the mean number of migrated cells in each group. Data are expressed as the mean ± standard error of the mean of three independent experiments. **p < 0.01, ****p < 0.0001. Figure S6. Original uncropped western blots from Figure 2e. Figure S7. The sarcoma cells and the normal dermal cells were irradiated with blue light (1.3 mW/cm2) for 48 h, and then mRNA expression of HO‐1 and oxidative stress induced growth inhibitor 1 (OSGIN1) was measured by qPCR. Data are expressed as the mean ± standard error of the mean of at least three independent experiments. *p < 0.05, **p < 0.01, ***p < 0.001. Figure S8. Density plots of flow cytometry analysis of apoptosis in NHDF cells after blue light irradiation with staurosporine treatment for 24 h as positive control. Figure S9. Original uncropped western blots from Figure 3c. Figure S10. Original uncropped western b [file CAM4-14-e70770-s002.zip › cam470770-sup-0007-supinfo_supplementary figure15-1.tif]

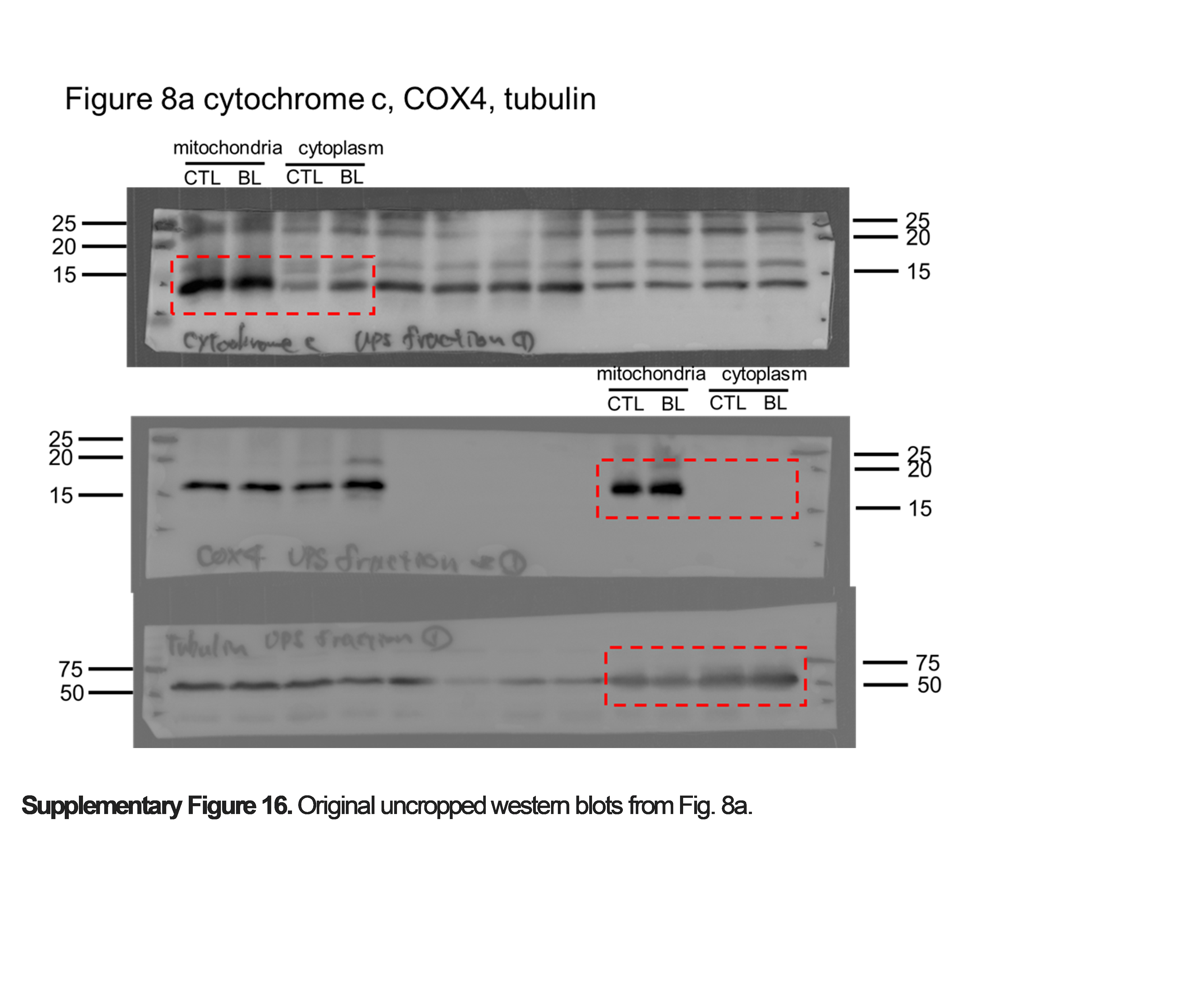

Supplement: Supplementary file 1 — Figure S1. Figure of a multi‐well plate placed in the LED device. Figure S2. The parameters of the LED light source used in these experiments. (a) Temperature of the culture medium in the plate with the LED device used. The LED light intensity used in this experiment (0.1, 0.6, and 1.3 mW/cm2) did not increase the temperature of the medium in a multi‐well plate nearly as much as in the non‐irradiated group (0 mW/cm2). (b) The light dose was calculated using the following formula: light dose (J/cm2) = light intensity (W/cm2) × irradiation time (seconds). Figure S3. Cell morphology of U‐2 OS, NCC‐UPS1‐C1, SW872, NCC‐MFS4‐C1, and NHDF cells with or without blue light (1.3 mW/cm2) for 48 h. Figure S4. Effects of blue LED irradiation on invasion in sarcoma cells. (a) Wound healing assay of sarcoma cells with continuous blue LED irradiation. Scale bar = 100 μm. (b) Quantification of the mean percentage of wound distance in each group. *p < 0.05, **p < 0.01, ***p < 0.001. Figure S5. Effects of blue LED irradiation on migration in sarcoma cells. (a) Migration abilities, as measured using Transwell filters. (b) Quantification of the mean number of migrated cells in each group. Data are expressed as the mean ± standard error of the mean of three independent experiments. **p < 0.01, ****p < 0.0001. Figure S6. Original uncropped western blots from Figure 2e. Figure S7. The sarcoma cells and the normal dermal cells were irradiated with blue light (1.3 mW/cm2) for 48 h, and then mRNA expression of HO‐1 and oxidative stress induced growth inhibitor 1 (OSGIN1) was measured by qPCR. Data are expressed as the mean ± standard error of the mean of at least three independent experiments. *p < 0.05, **p < 0.01, ***p < 0.001. Figure S8. Density plots of flow cytometry analysis of apoptosis in NHDF cells after blue light irradiation with staurosporine treatment for 24 h as positive control. Figure S9. Original uncropped western blots from Figure 3c. Figure S10. Original uncropped western b [file CAM4-14-e70770-s002.zip › cam470770-sup-0008-supinfo_supplementary figure16-1.tif]

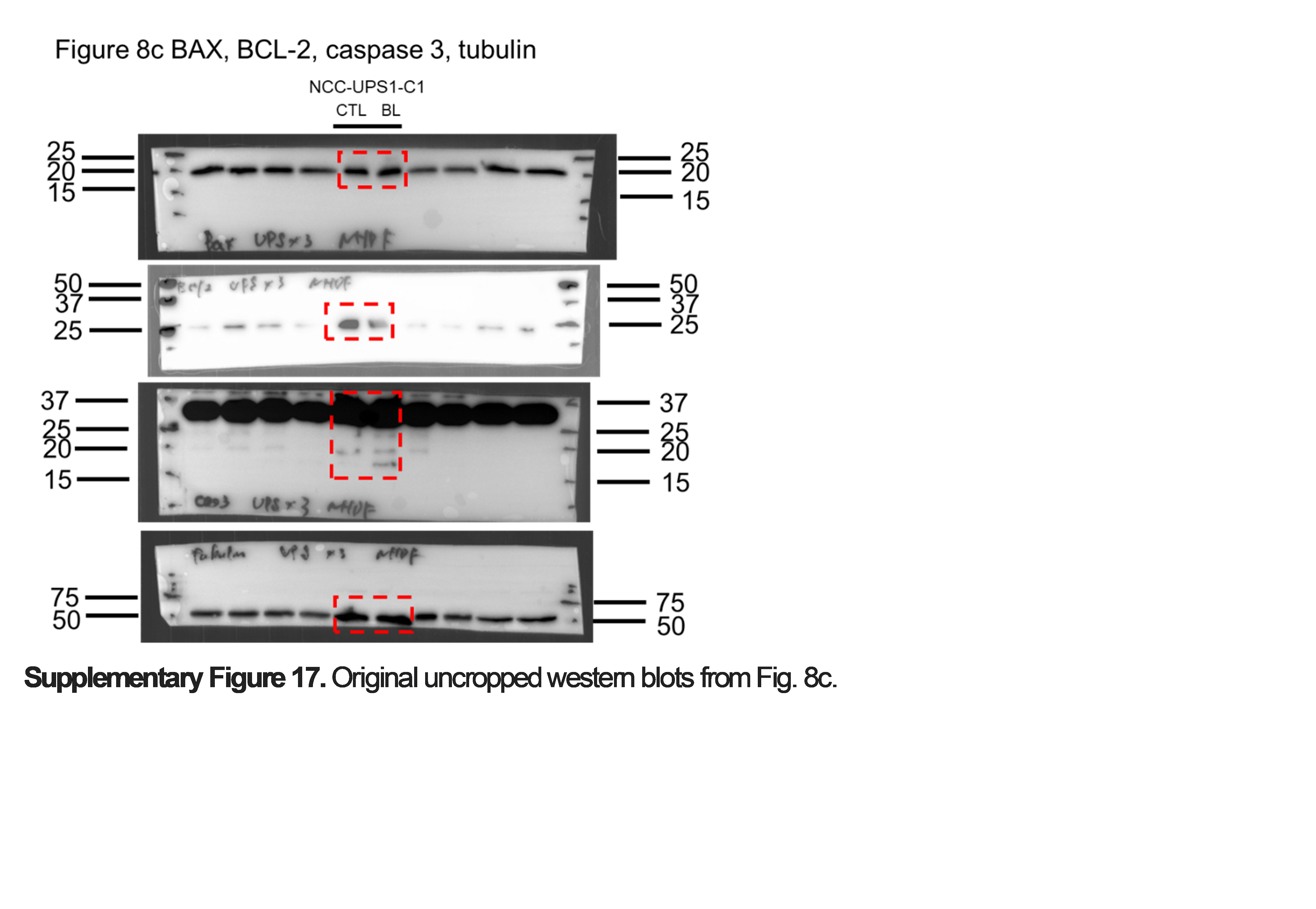

Supplement: Supplementary file 1 — Figure S1. Figure of a multi‐well plate placed in the LED device. Figure S2. The parameters of the LED light source used in these experiments. (a) Temperature of the culture medium in the plate with the LED device used. The LED light intensity used in this experiment (0.1, 0.6, and 1.3 mW/cm2) did not increase the temperature of the medium in a multi‐well plate nearly as much as in the non‐irradiated group (0 mW/cm2). (b) The light dose was calculated using the following formula: light dose (J/cm2) = light intensity (W/cm2) × irradiation time (seconds). Figure S3. Cell morphology of U‐2 OS, NCC‐UPS1‐C1, SW872, NCC‐MFS4‐C1, and NHDF cells with or without blue light (1.3 mW/cm2) for 48 h. Figure S4. Effects of blue LED irradiation on invasion in sarcoma cells. (a) Wound healing assay of sarcoma cells with continuous blue LED irradiation. Scale bar = 100 μm. (b) Quantification of the mean percentage of wound distance in each group. *p < 0.05, **p < 0.01, ***p < 0.001. Figure S5. Effects of blue LED irradiation on migration in sarcoma cells. (a) Migration abilities, as measured using Transwell filters. (b) Quantification of the mean number of migrated cells in each group. Data are expressed as the mean ± standard error of the mean of three independent experiments. **p < 0.01, ****p < 0.0001. Figure S6. Original uncropped western blots from Figure 2e. Figure S7. The sarcoma cells and the normal dermal cells were irradiated with blue light (1.3 mW/cm2) for 48 h, and then mRNA expression of HO‐1 and oxidative stress induced growth inhibitor 1 (OSGIN1) was measured by qPCR. Data are expressed as the mean ± standard error of the mean of at least three independent experiments. *p < 0.05, **p < 0.01, ***p < 0.001. Figure S8. Density plots of flow cytometry analysis of apoptosis in NHDF cells after blue light irradiation with staurosporine treatment for 24 h as positive control. Figure S9. Original uncropped western blots from Figure 3c. Figure S10. Original uncropped western b [file CAM4-14-e70770-s002.zip › cam470770-sup-0009-supinfo_supplementary figure17-1.tif]

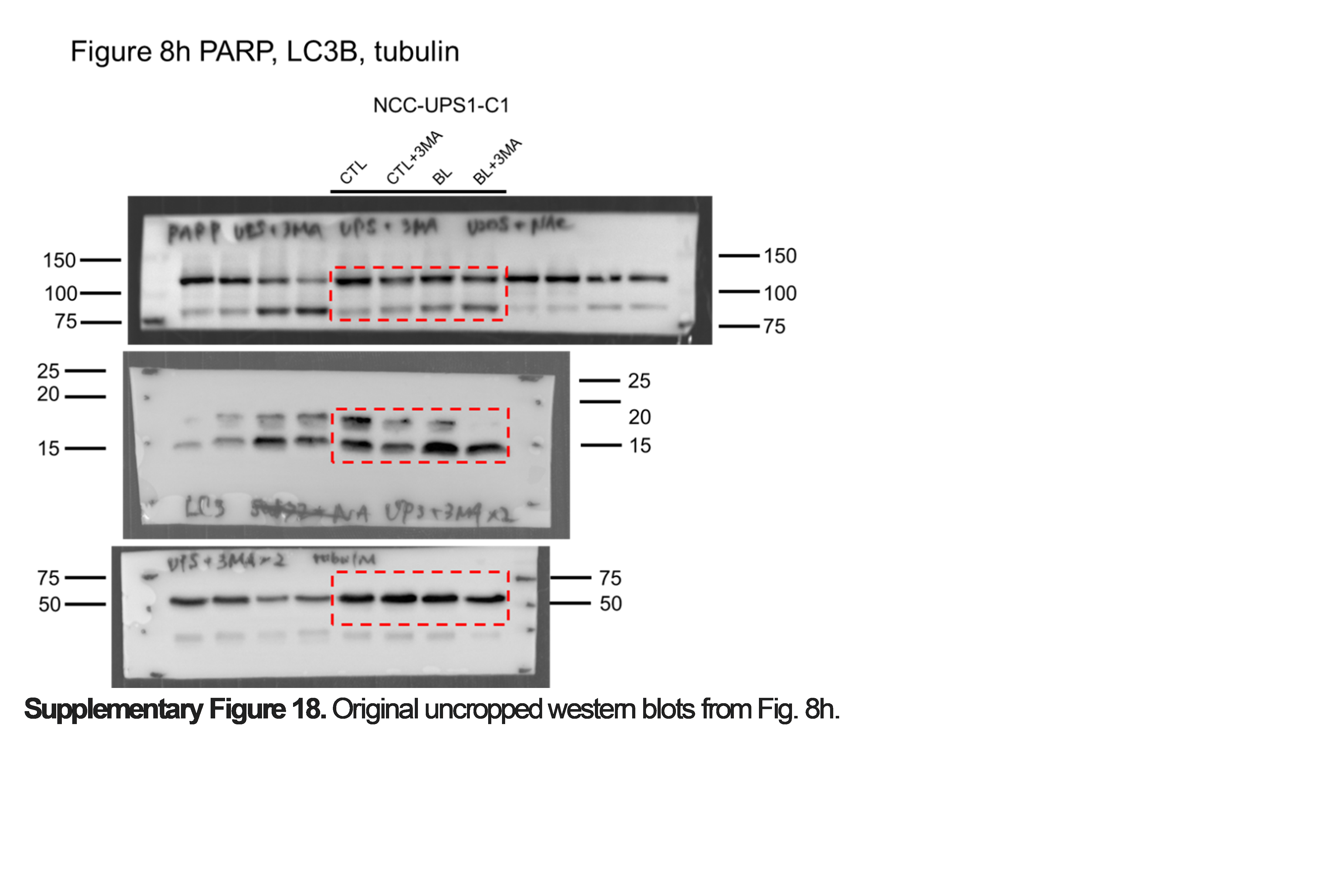

Supplement: Supplementary file 1 — Figure S1. Figure of a multi‐well plate placed in the LED device. Figure S2. The parameters of the LED light source used in these experiments. (a) Temperature of the culture medium in the plate with the LED device used. The LED light intensity used in this experiment (0.1, 0.6, and 1.3 mW/cm2) did not increase the temperature of the medium in a multi‐well plate nearly as much as in the non‐irradiated group (0 mW/cm2). (b) The light dose was calculated using the following formula: light dose (J/cm2) = light intensity (W/cm2) × irradiation time (seconds). Figure S3. Cell morphology of U‐2 OS, NCC‐UPS1‐C1, SW872, NCC‐MFS4‐C1, and NHDF cells with or without blue light (1.3 mW/cm2) for 48 h. Figure S4. Effects of blue LED irradiation on invasion in sarcoma cells. (a) Wound healing assay of sarcoma cells with continuous blue LED irradiation. Scale bar = 100 μm. (b) Quantification of the mean percentage of wound distance in each group. *p < 0.05, **p < 0.01, ***p < 0.001. Figure S5. Effects of blue LED irradiation on migration in sarcoma cells. (a) Migration abilities, as measured using Transwell filters. (b) Quantification of the mean number of migrated cells in each group. Data are expressed as the mean ± standard error of the mean of three independent experiments. **p < 0.01, ****p < 0.0001. Figure S6. Original uncropped western blots from Figure 2e. Figure S7. The sarcoma cells and the normal dermal cells were irradiated with blue light (1.3 mW/cm2) for 48 h, and then mRNA expression of HO‐1 and oxidative stress induced growth inhibitor 1 (OSGIN1) was measured by qPCR. Data are expressed as the mean ± standard error of the mean of at least three independent experiments. *p < 0.05, **p < 0.01, ***p < 0.001. Figure S8. Density plots of flow cytometry analysis of apoptosis in NHDF cells after blue light irradiation with staurosporine treatment for 24 h as positive control. Figure S9. Original uncropped western blots from Figure 3c. Figure S10. Original uncropped western b [file CAM4-14-e70770-s002.zip › cam470770-sup-0010-supinfo_supplementary figure18-1.tif]

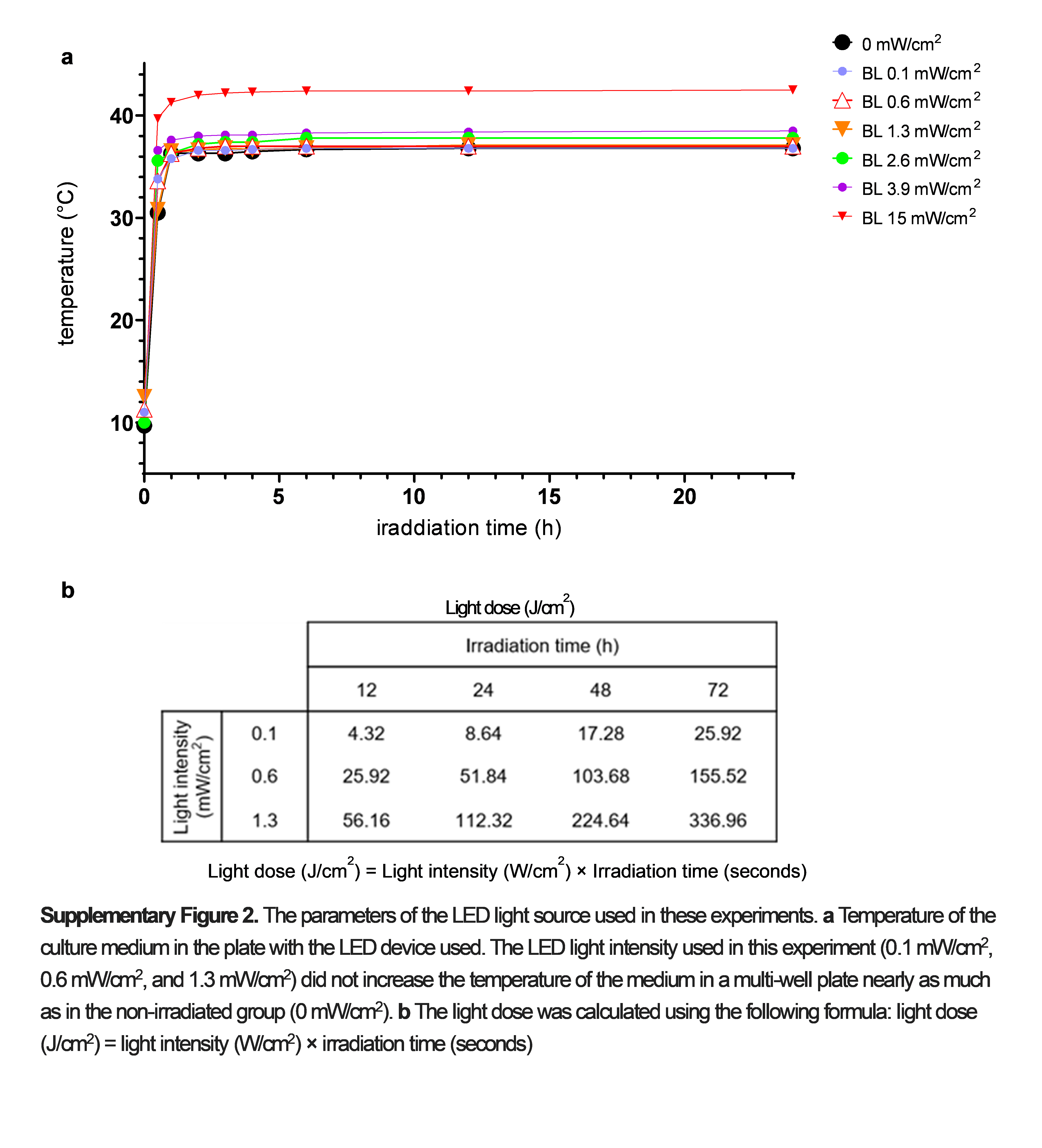

Supplement: Supplementary file 1 — Figure S1. Figure of a multi‐well plate placed in the LED device. Figure S2. The parameters of the LED light source used in these experiments. (a) Temperature of the culture medium in the plate with the LED device used. The LED light intensity used in this experiment (0.1, 0.6, and 1.3 mW/cm2) did not increase the temperature of the medium in a multi‐well plate nearly as much as in the non‐irradiated group (0 mW/cm2). (b) The light dose was calculated using the following formula: light dose (J/cm2) = light intensity (W/cm2) × irradiation time (seconds). Figure S3. Cell morphology of U‐2 OS, NCC‐UPS1‐C1, SW872, NCC‐MFS4‐C1, and NHDF cells with or without blue light (1.3 mW/cm2) for 48 h. Figure S4. Effects of blue LED irradiation on invasion in sarcoma cells. (a) Wound healing assay of sarcoma cells with continuous blue LED irradiation. Scale bar = 100 μm. (b) Quantification of the mean percentage of wound distance in each group. *p < 0.05, **p < 0.01, ***p < 0.001. Figure S5. Effects of blue LED irradiation on migration in sarcoma cells. (a) Migration abilities, as measured using Transwell filters. (b) Quantification of the mean number of migrated cells in each group. Data are expressed as the mean ± standard error of the mean of three independent experiments. **p < 0.01, ****p < 0.0001. Figure S6. Original uncropped western blots from Figure 2e. Figure S7. The sarcoma cells and the normal dermal cells were irradiated with blue light (1.3 mW/cm2) for 48 h, and then mRNA expression of HO‐1 and oxidative stress induced growth inhibitor 1 (OSGIN1) was measured by qPCR. Data are expressed as the mean ± standard error of the mean of at least three independent experiments. *p < 0.05, **p < 0.01, ***p < 0.001. Figure S8. Density plots of flow cytometry analysis of apoptosis in NHDF cells after blue light irradiation with staurosporine treatment for 24 h as positive control. Figure S9. Original uncropped western blots from Figure 3c. Figure S10. Original uncropped western b [file CAM4-14-e70770-s002.zip › cam470770-sup-0011-supinfo_supplementary figure2-1.tif]

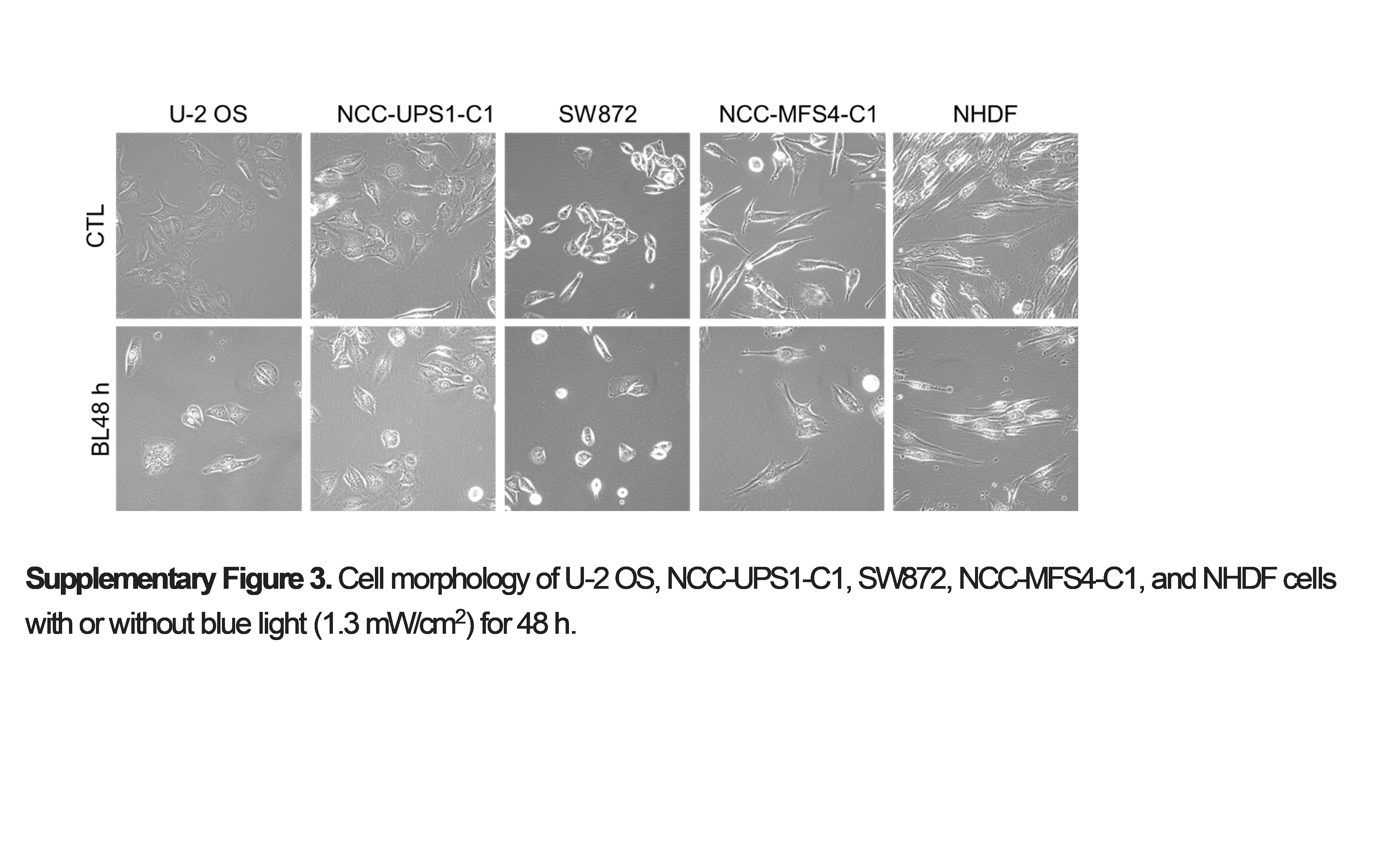

Supplement: Supplementary file 1 — Figure S1. Figure of a multi‐well plate placed in the LED device. Figure S2. The parameters of the LED light source used in these experiments. (a) Temperature of the culture medium in the plate with the LED device used. The LED light intensity used in this experiment (0.1, 0.6, and 1.3 mW/cm2) did not increase the temperature of the medium in a multi‐well plate nearly as much as in the non‐irradiated group (0 mW/cm2). (b) The light dose was calculated using the following formula: light dose (J/cm2) = light intensity (W/cm2) × irradiation time (seconds). Figure S3. Cell morphology of U‐2 OS, NCC‐UPS1‐C1, SW872, NCC‐MFS4‐C1, and NHDF cells with or without blue light (1.3 mW/cm2) for 48 h. Figure S4. Effects of blue LED irradiation on invasion in sarcoma cells. (a) Wound healing assay of sarcoma cells with continuous blue LED irradiation. Scale bar = 100 μm. (b) Quantification of the mean percentage of wound distance in each group. *p < 0.05, **p < 0.01, ***p < 0.001. Figure S5. Effects of blue LED irradiation on migration in sarcoma cells. (a) Migration abilities, as measured using Transwell filters. (b) Quantification of the mean number of migrated cells in each group. Data are expressed as the mean ± standard error of the mean of three independent experiments. **p < 0.01, ****p < 0.0001. Figure S6. Original uncropped western blots from Figure 2e. Figure S7. The sarcoma cells and the normal dermal cells were irradiated with blue light (1.3 mW/cm2) for 48 h, and then mRNA expression of HO‐1 and oxidative stress induced growth inhibitor 1 (OSGIN1) was measured by qPCR. Data are expressed as the mean ± standard error of the mean of at least three independent experiments. *p < 0.05, **p < 0.01, ***p < 0.001. Figure S8. Density plots of flow cytometry analysis of apoptosis in NHDF cells after blue light irradiation with staurosporine treatment for 24 h as positive control. Figure S9. Original uncropped western blots from Figure 3c. Figure S10. Original uncropped western b [file CAM4-14-e70770-s002.zip › cam470770-sup-0012-supinfo_supplementary figure3-1.tif]

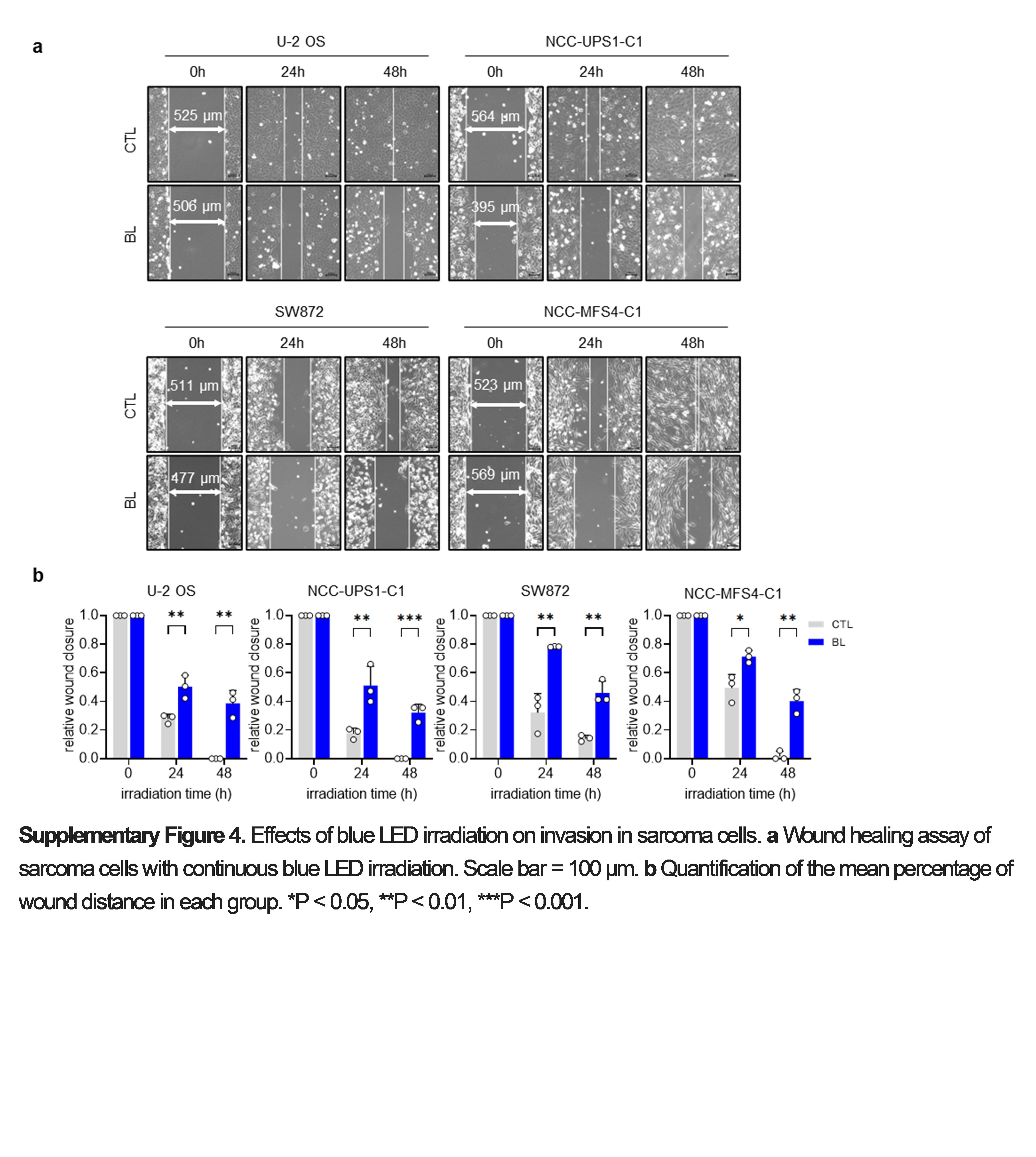

Supplement: Supplementary file 1 — Figure S1. Figure of a multi‐well plate placed in the LED device. Figure S2. The parameters of the LED light source used in these experiments. (a) Temperature of the culture medium in the plate with the LED device used. The LED light intensity used in this experiment (0.1, 0.6, and 1.3 mW/cm2) did not increase the temperature of the medium in a multi‐well plate nearly as much as in the non‐irradiated group (0 mW/cm2). (b) The light dose was calculated using the following formula: light dose (J/cm2) = light intensity (W/cm2) × irradiation time (seconds). Figure S3. Cell morphology of U‐2 OS, NCC‐UPS1‐C1, SW872, NCC‐MFS4‐C1, and NHDF cells with or without blue light (1.3 mW/cm2) for 48 h. Figure S4. Effects of blue LED irradiation on invasion in sarcoma cells. (a) Wound healing assay of sarcoma cells with continuous blue LED irradiation. Scale bar = 100 μm. (b) Quantification of the mean percentage of wound distance in each group. *p < 0.05, **p < 0.01, ***p < 0.001. Figure S5. Effects of blue LED irradiation on migration in sarcoma cells. (a) Migration abilities, as measured using Transwell filters. (b) Quantification of the mean number of migrated cells in each group. Data are expressed as the mean ± standard error of the mean of three independent experiments. **p < 0.01, ****p < 0.0001. Figure S6. Original uncropped western blots from Figure 2e. Figure S7. The sarcoma cells and the normal dermal cells were irradiated with blue light (1.3 mW/cm2) for 48 h, and then mRNA expression of HO‐1 and oxidative stress induced growth inhibitor 1 (OSGIN1) was measured by qPCR. Data are expressed as the mean ± standard error of the mean of at least three independent experiments. *p < 0.05, **p < 0.01, ***p < 0.001. Figure S8. Density plots of flow cytometry analysis of apoptosis in NHDF cells after blue light irradiation with staurosporine treatment for 24 h as positive control. Figure S9. Original uncropped western blots from Figure 3c. Figure S10. Original uncropped western b [file CAM4-14-e70770-s002.zip › cam470770-sup-0013-supinfo_supplementary figure4-1.tif]

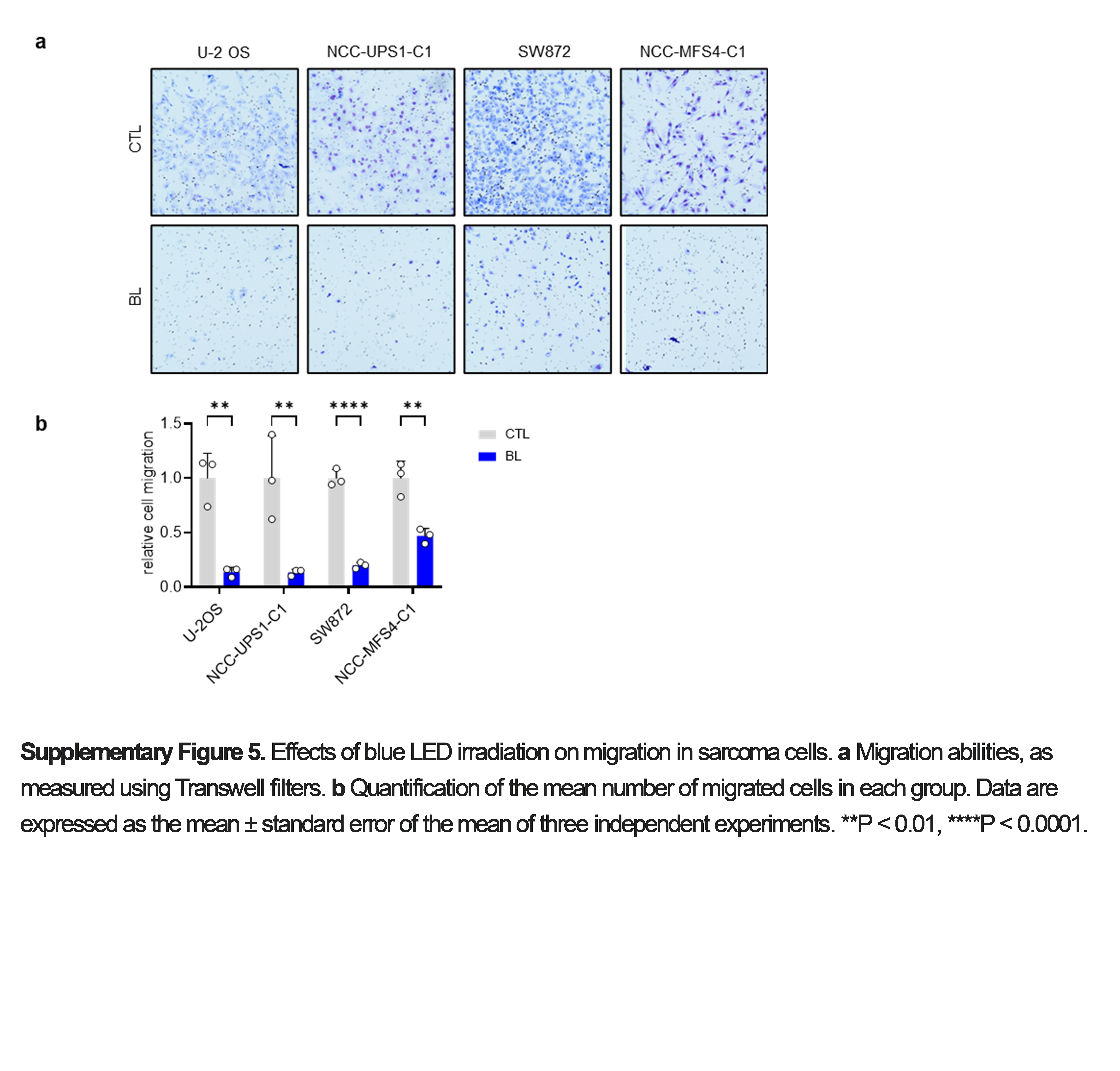

Supplement: Supplementary file 1 — Figure S1. Figure of a multi‐well plate placed in the LED device. Figure S2. The parameters of the LED light source used in these experiments. (a) Temperature of the culture medium in the plate with the LED device used. The LED light intensity used in this experiment (0.1, 0.6, and 1.3 mW/cm2) did not increase the temperature of the medium in a multi‐well plate nearly as much as in the non‐irradiated group (0 mW/cm2). (b) The light dose was calculated using the following formula: light dose (J/cm2) = light intensity (W/cm2) × irradiation time (seconds). Figure S3. Cell morphology of U‐2 OS, NCC‐UPS1‐C1, SW872, NCC‐MFS4‐C1, and NHDF cells with or without blue light (1.3 mW/cm2) for 48 h. Figure S4. Effects of blue LED irradiation on invasion in sarcoma cells. (a) Wound healing assay of sarcoma cells with continuous blue LED irradiation. Scale bar = 100 μm. (b) Quantification of the mean percentage of wound distance in each group. *p < 0.05, **p < 0.01, ***p < 0.001. Figure S5. Effects of blue LED irradiation on migration in sarcoma cells. (a) Migration abilities, as measured using Transwell filters. (b) Quantification of the mean number of migrated cells in each group. Data are expressed as the mean ± standard error of the mean of three independent experiments. **p < 0.01, ****p < 0.0001. Figure S6. Original uncropped western blots from Figure 2e. Figure S7. The sarcoma cells and the normal dermal cells were irradiated with blue light (1.3 mW/cm2) for 48 h, and then mRNA expression of HO‐1 and oxidative stress induced growth inhibitor 1 (OSGIN1) was measured by qPCR. Data are expressed as the mean ± standard error of the mean of at least three independent experiments. *p < 0.05, **p < 0.01, ***p < 0.001. Figure S8. Density plots of flow cytometry analysis of apoptosis in NHDF cells after blue light irradiation with staurosporine treatment for 24 h as positive control. Figure S9. Original uncropped western blots from Figure 3c. Figure S10. Original uncropped western b [file CAM4-14-e70770-s002.zip › cam470770-sup-0014-supinfo_supplementary figure5-1.tif]

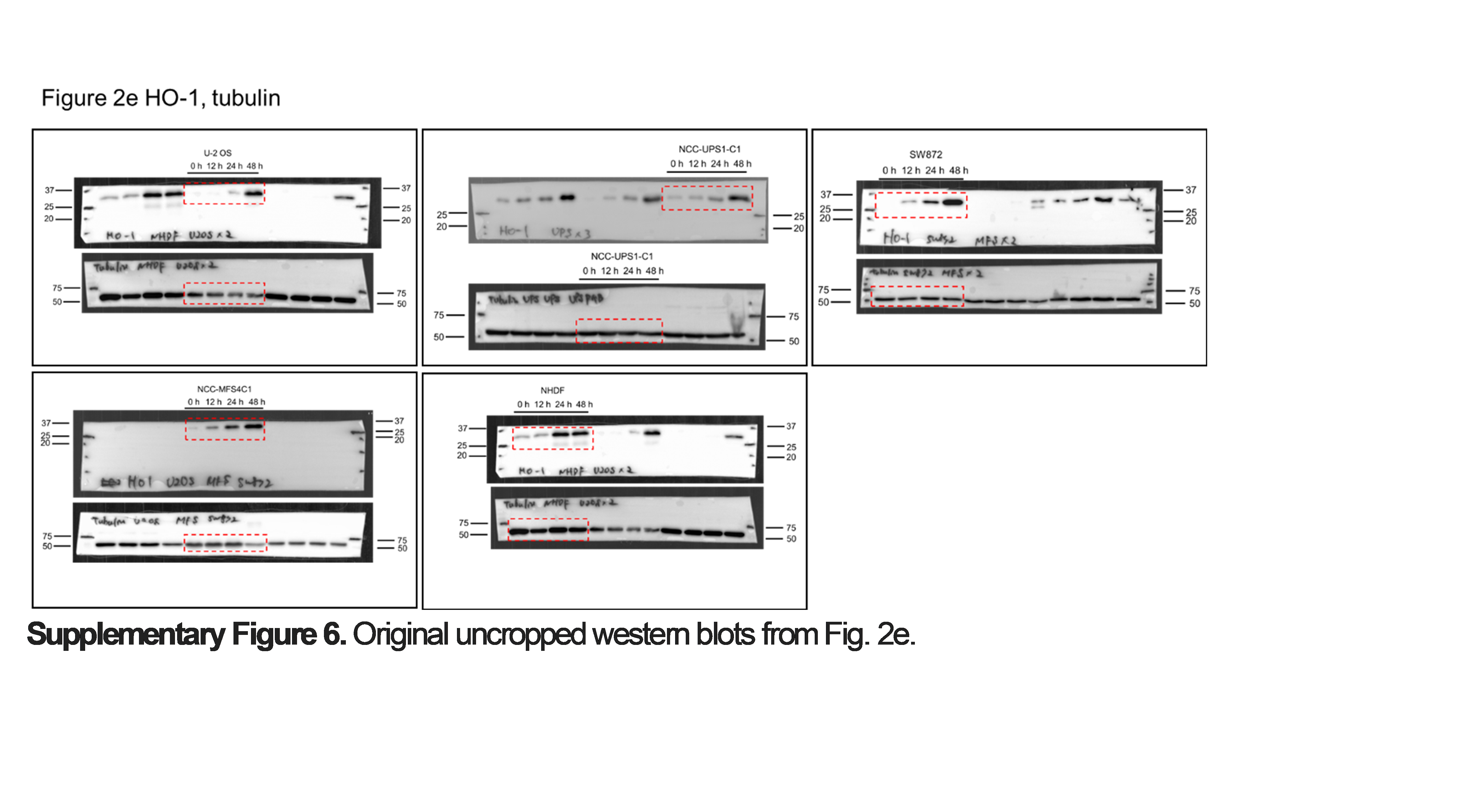

Supplement: Supplementary file 1 — Figure S1. Figure of a multi‐well plate placed in the LED device. Figure S2. The parameters of the LED light source used in these experiments. (a) Temperature of the culture medium in the plate with the LED device used. The LED light intensity used in this experiment (0.1, 0.6, and 1.3 mW/cm2) did not increase the temperature of the medium in a multi‐well plate nearly as much as in the non‐irradiated group (0 mW/cm2). (b) The light dose was calculated using the following formula: light dose (J/cm2) = light intensity (W/cm2) × irradiation time (seconds). Figure S3. Cell morphology of U‐2 OS, NCC‐UPS1‐C1, SW872, NCC‐MFS4‐C1, and NHDF cells with or without blue light (1.3 mW/cm2) for 48 h. Figure S4. Effects of blue LED irradiation on invasion in sarcoma cells. (a) Wound healing assay of sarcoma cells with continuous blue LED irradiation. Scale bar = 100 μm. (b) Quantification of the mean percentage of wound distance in each group. *p < 0.05, **p < 0.01, ***p < 0.001. Figure S5. Effects of blue LED irradiation on migration in sarcoma cells. (a) Migration abilities, as measured using Transwell filters. (b) Quantification of the mean number of migrated cells in each group. Data are expressed as the mean ± standard error of the mean of three independent experiments. **p < 0.01, ****p < 0.0001. Figure S6. Original uncropped western blots from Figure 2e. Figure S7. The sarcoma cells and the normal dermal cells were irradiated with blue light (1.3 mW/cm2) for 48 h, and then mRNA expression of HO‐1 and oxidative stress induced growth inhibitor 1 (OSGIN1) was measured by qPCR. Data are expressed as the mean ± standard error of the mean of at least three independent experiments. *p < 0.05, **p < 0.01, ***p < 0.001. Figure S8. Density plots of flow cytometry analysis of apoptosis in NHDF cells after blue light irradiation with staurosporine treatment for 24 h as positive control. Figure S9. Original uncropped western blots from Figure 3c. Figure S10. Original uncropped western b [file CAM4-14-e70770-s002.zip › cam470770-sup-0015-supinfo_supplementary figure6-1.tif]

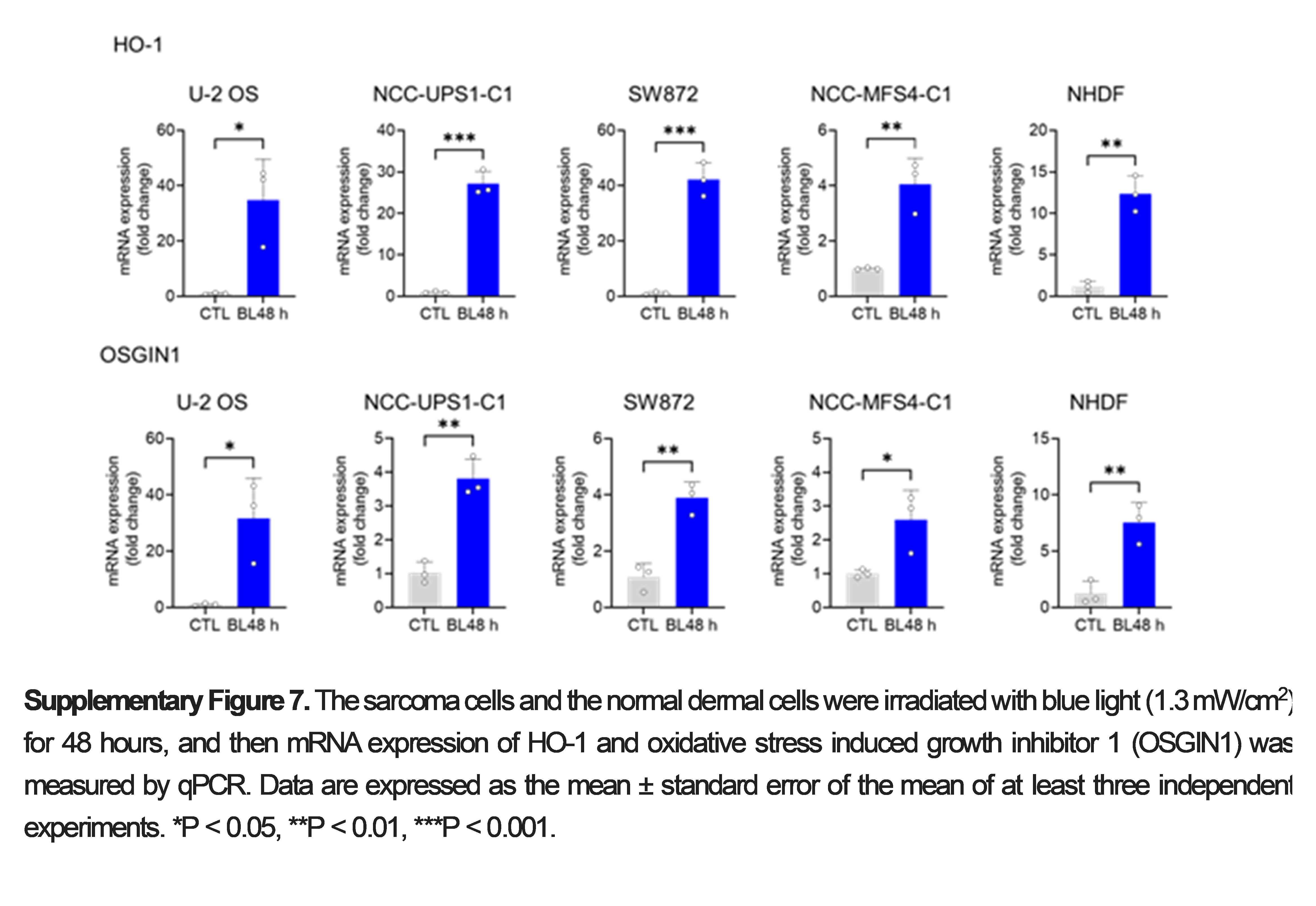

Supplement: Supplementary file 1 — Figure S1. Figure of a multi‐well plate placed in the LED device. Figure S2. The parameters of the LED light source used in these experiments. (a) Temperature of the culture medium in the plate with the LED device used. The LED light intensity used in this experiment (0.1, 0.6, and 1.3 mW/cm2) did not increase the temperature of the medium in a multi‐well plate nearly as much as in the non‐irradiated group (0 mW/cm2). (b) The light dose was calculated using the following formula: light dose (J/cm2) = light intensity (W/cm2) × irradiation time (seconds). Figure S3. Cell morphology of U‐2 OS, NCC‐UPS1‐C1, SW872, NCC‐MFS4‐C1, and NHDF cells with or without blue light (1.3 mW/cm2) for 48 h. Figure S4. Effects of blue LED irradiation on invasion in sarcoma cells. (a) Wound healing assay of sarcoma cells with continuous blue LED irradiation. Scale bar = 100 μm. (b) Quantification of the mean percentage of wound distance in each group. *p < 0.05, **p < 0.01, ***p < 0.001. Figure S5. Effects of blue LED irradiation on migration in sarcoma cells. (a) Migration abilities, as measured using Transwell filters. (b) Quantification of the mean number of migrated cells in each group. Data are expressed as the mean ± standard error of the mean of three independent experiments. **p < 0.01, ****p < 0.0001. Figure S6. Original uncropped western blots from Figure 2e. Figure S7. The sarcoma cells and the normal dermal cells were irradiated with blue light (1.3 mW/cm2) for 48 h, and then mRNA expression of HO‐1 and oxidative stress induced growth inhibitor 1 (OSGIN1) was measured by qPCR. Data are expressed as the mean ± standard error of the mean of at least three independent experiments. *p < 0.05, **p < 0.01, ***p < 0.001. Figure S8. Density plots of flow cytometry analysis of apoptosis in NHDF cells after blue light irradiation with staurosporine treatment for 24 h as positive control. Figure S9. Original uncropped western blots from Figure 3c. Figure S10. Original uncropped western b [file CAM4-14-e70770-s002.zip › cam470770-sup-0016-supinfo_supplementary figure7-1.tif]

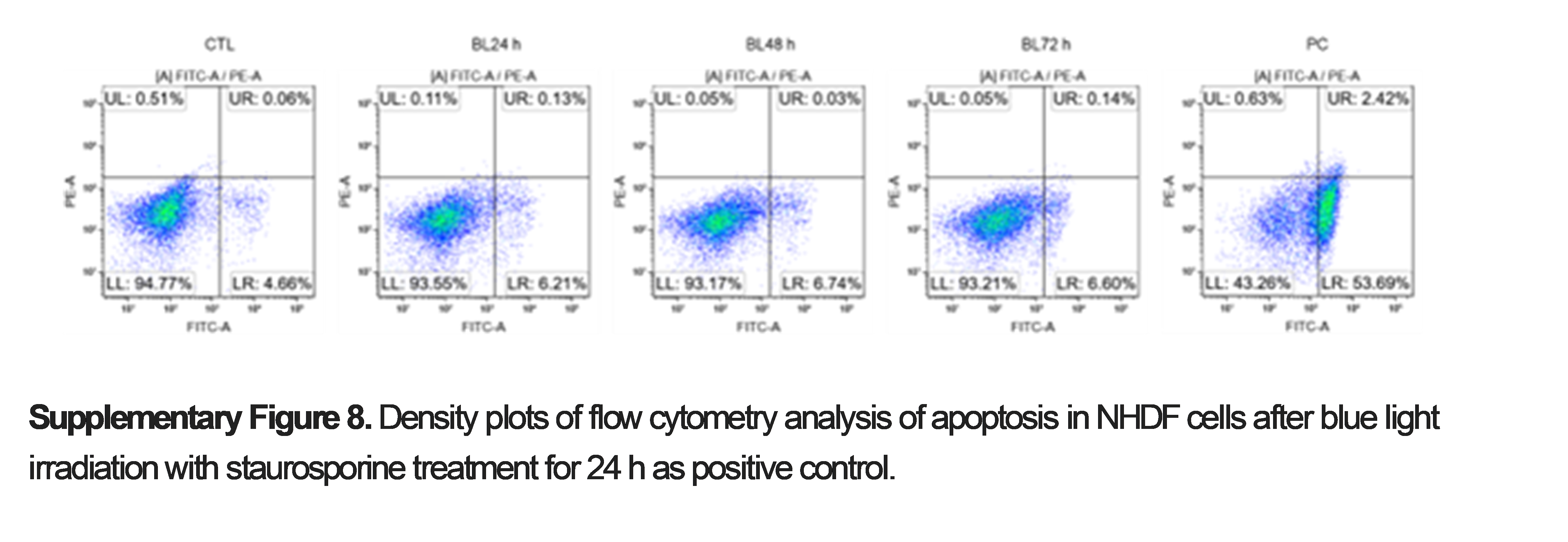

Supplement: Supplementary file 1 — Figure S1. Figure of a multi‐well plate placed in the LED device. Figure S2. The parameters of the LED light source used in these experiments. (a) Temperature of the culture medium in the plate with the LED device used. The LED light intensity used in this experiment (0.1, 0.6, and 1.3 mW/cm2) did not increase the temperature of the medium in a multi‐well plate nearly as much as in the non‐irradiated group (0 mW/cm2). (b) The light dose was calculated using the following formula: light dose (J/cm2) = light intensity (W/cm2) × irradiation time (seconds). Figure S3. Cell morphology of U‐2 OS, NCC‐UPS1‐C1, SW872, NCC‐MFS4‐C1, and NHDF cells with or without blue light (1.3 mW/cm2) for 48 h. Figure S4. Effects of blue LED irradiation on invasion in sarcoma cells. (a) Wound healing assay of sarcoma cells with continuous blue LED irradiation. Scale bar = 100 μm. (b) Quantification of the mean percentage of wound distance in each group. *p < 0.05, **p < 0.01, ***p < 0.001. Figure S5. Effects of blue LED irradiation on migration in sarcoma cells. (a) Migration abilities, as measured using Transwell filters. (b) Quantification of the mean number of migrated cells in each group. Data are expressed as the mean ± standard error of the mean of three independent experiments. **p < 0.01, ****p < 0.0001. Figure S6. Original uncropped western blots from Figure 2e. Figure S7. The sarcoma cells and the normal dermal cells were irradiated with blue light (1.3 mW/cm2) for 48 h, and then mRNA expression of HO‐1 and oxidative stress induced growth inhibitor 1 (OSGIN1) was measured by qPCR. Data are expressed as the mean ± standard error of the mean of at least three independent experiments. *p < 0.05, **p < 0.01, ***p < 0.001. Figure S8. Density plots of flow cytometry analysis of apoptosis in NHDF cells after blue light irradiation with staurosporine treatment for 24 h as positive control. Figure S9. Original uncropped western blots from Figure 3c. Figure S10. Original uncropped western b [file CAM4-14-e70770-s002.zip › cam470770-sup-0017-supinfo_supplementary figure8-1.tif]

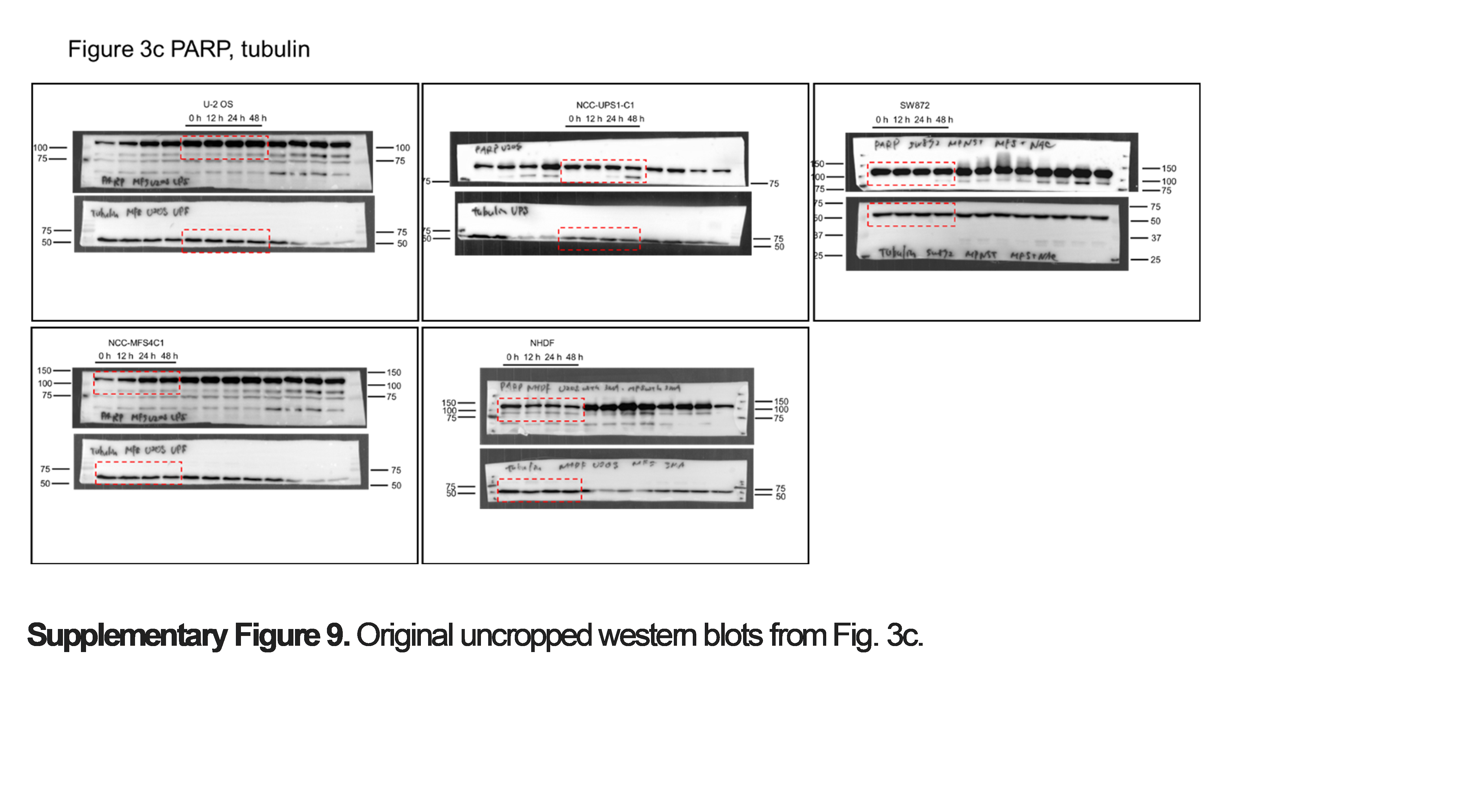

Supplement: Supplementary file 1 — Figure S1. Figure of a multi‐well plate placed in the LED device. Figure S2. The parameters of the LED light source used in these experiments. (a) Temperature of the culture medium in the plate with the LED device used. The LED light intensity used in this experiment (0.1, 0.6, and 1.3 mW/cm2) did not increase the temperature of the medium in a multi‐well plate nearly as much as in the non‐irradiated group (0 mW/cm2). (b) The light dose was calculated using the following formula: light dose (J/cm2) = light intensity (W/cm2) × irradiation time (seconds). Figure S3. Cell morphology of U‐2 OS, NCC‐UPS1‐C1, SW872, NCC‐MFS4‐C1, and NHDF cells with or without blue light (1.3 mW/cm2) for 48 h. Figure S4. Effects of blue LED irradiation on invasion in sarcoma cells. (a) Wound healing assay of sarcoma cells with continuous blue LED irradiation. Scale bar = 100 μm. (b) Quantification of the mean percentage of wound distance in each group. *p < 0.05, **p < 0.01, ***p < 0.001. Figure S5. Effects of blue LED irradiation on migration in sarcoma cells. (a) Migration abilities, as measured using Transwell filters. (b) Quantification of the mean number of migrated cells in each group. Data are expressed as the mean ± standard error of the mean of three independent experiments. **p < 0.01, ****p < 0.0001. Figure S6. Original uncropped western blots from Figure 2e. Figure S7. The sarcoma cells and the normal dermal cells were irradiated with blue light (1.3 mW/cm2) for 48 h, and then mRNA expression of HO‐1 and oxidative stress induced growth inhibitor 1 (OSGIN1) was measured by qPCR. Data are expressed as the mean ± standard error of the mean of at least three independent experiments. *p < 0.05, **p < 0.01, ***p < 0.001. Figure S8. Density plots of flow cytometry analysis of apoptosis in NHDF cells after blue light irradiation with staurosporine treatment for 24 h as positive control. Figure S9. Original uncropped western blots from Figure 3c. Figure S10. Original uncropped western b [file CAM4-14-e70770-s002.zip › cam470770-sup-0018-supinfo_supplementary figure9-1.tif]
