# Supplementary material for: Blue Light Emitting Diode Suppresses Sarcoma Cell Proliferation via the Endogenous Apoptotic Pathway Without Damaging Normal Cells
Source: Cancer Med. 2025 Mar 24;14(6):e70770. doi: 10.1002/cam4.70770 (PMC11931449; doi:10.1002/cam4.70770)
Supplement: Supplementary file 2 — Table S1. Reagents used in this study. Table S2. Sequence of primers used for qPCR. [file CAM4-14-e70770-s001.docx]

Supplementary Information for

**Blue light emitting diode suppresses sarcoma cell proliferation via the endogenous apoptotic pathway without damaging normal cells**

**Contents**

Supplementary Tables 1–2

**Supplementary Table 1.** Reagents used in this study.

| **Reagent** | **Source** | **Catalog number** |
| --- | --- | --- |
| Seahorse XF Cell Mito Stress Test Kit | Agilent Technologies (Santa Clara, CA) | 103010-100 |
| DMEM Assay Medium Pack | Agilent Technologies (Santa Clara, CA) | 103680-100 |
| 2-Mercaptoethanol | Bio-Rad (Hercules, CA) | 1610710 |
| 10× Tris/Glycine/SDS | Bio-Rad (Hercules, CA) | 1610732 |
| 10× Tris/Glycine | Bio-Rad (Hercules, CA) | 1610734 |
| 10× TBS | Bio-Rad (Hercules, CA) | 1706435 |
| 10% Tween 20 | Bio-Rad (Hercules, CA) | 1610781 |
| 30% Acrylamide/Bis Solution | Bio-Rad (Hercules, CA) | 1610158 |
| Resolving Gel Buffer for PAGE | Bio-Rad (Hercules, CA) | 1610798 |
| Stacking Gel Buffer for PAGE | Bio-Rad (Hercules, CA) | 1610799 |
| Ammonium Persulfate (APS) | Bio-Rad (Hercules, CA) | 1610700 |
| TEMED | Bio-Rad (Hercules, CA) | 1610800 |
| Precision Plus Protein Dual Color Standards | Bio-Rad (Hercules, CA) | 1610374 |
| Precision Plus Protein All Blue Prestained | Bio-Rad (Hercules, CA) | 1610373 |
| iScript Advanced cDNA Synthesis kit | Bio-Rad (Hercules, CA) | 1725038 |
| EvaGreen 20× in Water | Biotium (Fremont, CA) | 31000 |
| PARP Antibody | Cell Signaling Technology (Danvers, MA) | 9542 |
| HO-1 (E3F4S) | Cell Signaling Technology (Danvers, MA) | 43966 |
| LC3B Antibody | Cell Signaling Technology (Danvers, MA) | 2775 |
| BCL-2 Antibody | Cell Signaling Technology (Danvers, MA) | 4223 |
| BAX Antibody | Cell Signaling Technology (Danvers, MA) | 2772 |
| Caspase-3 Antibody | Cell Signaling Technology (Danvers, MA) | 9662 |
| α/β-Tubulin Antibody | Cell Signaling Technology (Danvers, MA) | 2148 |
| Anti-rabbit IgG, HRP-linked Antibody | Cell Signaling Technology (Danvers, MA) | 7074S |
| ECL Prime Western Blotting Detection Reagent | Cytiva (Tokyo, Japan) | RPN2232 |
| Cell Counting Kit-8 | Dojindo (Kumamoto, Japan) | 341-07624 |
| JC-1 MitoMP Detection Kit | Dojindo (Kumamoto, Japan) | MT09 |
| CYTO-ID Autophagy Detection Kit | Enzo Life Sciences (Farmingdale, NY) | ENZ-KIT175-0200 |
| ApopTag Plus Peroxidase In Situ Apoptosis Kit | Millipore (Burlington, MA) | S7101 |
| 4%-Paraformaldehyde Phosphate Buffer Solution | Nakalai (Kyoto, Japan) | 09154-56 |
| Phosphate-Buffered Saline | Santa Cruz Biotechnology (Santa Cruz, CA) | |
| 3-Methyladenine | Selleckchem (Houston, TX) | 5142-23-4 |
| Dulbecco’s Modified Eagle’s Medium | Sigma-Aldrich (St. Louis, MO) | D6046 |
| Fetal Bovine Serum | Sigma-Aldrich (St. Louis, MO) | F7524 |
| Penicillin-Streptomycin | Sigma-Aldrich (St. Louis, MO) | D6046 |
| Crystal Violet Solution | Sigma-Aldrich (St. Louis, MO) | V5265 |
| N-Acetyl-L-cysteine | Sigma-Aldrich (St. Louis, MO) | A7250 |
| Protease Inhibitor Cocktail | Sigma-Aldrich (St. Louis, MO) | I3786 |
| Phosphatase Inhibitor Cocktail | Sigma-Aldrich (St. Louis, MO) | P0044 |
| Reference Dye for Quantitative PCR 100×, solution | Sigma-Aldrich (St. Louis, MO) | R4526 |
| CELLBANKER 1 | Takara (Shiga, Japan) | CB011 |
| BCA Protein Assay Kit | Takara (Shiga, Japan) | T9300A |
| Trypsin-EDTA (0.05%) | Thermo Fisher Scientific (Waltham, MA) | 25300062 |
| Dead Cell Apoptosis Kit with Annexin V-FITC and PI | Thermo Fisher Scientific (Waltham, MA) | V13242 |
| RIPA Lysis and Extraction Buffer | Thermo Fisher Scientific (Waltham, MA) | 89900 |
| CellROX Orange Flow Cytometry Assay Kit | Thermo Fisher Scientific (Waltham, MA) | C10493 |
| MitoSOX Red Mitochondrial Superoxide Indicator | Thermo Fisher Scientific (Waltham, MA) | M36008 |
| Hanks′ Balanced Salt Solution | Thermo Fisher Scientific (Waltham, MA) | 14025092 |
| Power SYBR Green PCR Master Mix | Thermo Fisher Scientific (Waltham, MA) | 4368706 |
| Hoechst 33342 | Thermo Fisher Scientific (Waltham, MA) | H3570 |
| Methanol | Wako (Osaka, Japan) | 137-01823 |
| Sodium Dodecyl Sulfate | Wako (Osaka, Japan) | 194-13985 |
| Cell Fractionation Kit | abcam (Cambridge, UK) | ab109719 |

**Supplementary Table 2.** Sequence of primers used for qPCR.

| **Gene** | **Forward primer (5'-3')** | **Reverse primer (5'-3')** |
| --- | --- | --- |
| HO-1 | ATTTCAGAAGGGCCAGGTGA | GGAAGTAGACAGGGGCGAAGA |
| OSGIN1 | GCAGCAGATGATGCGTGAC | GGAGCCGATGAGGACGAG |
| 18s | CAGAAGGATGTAAAGGATGG | TATTTCTTCTTGGACACACC |
